# Spontaneous Prophage Induction Contributes to the Production of Membrane Vesicles by the Gram-Positive Bacterium *Lacticaseibacillus casei* BL23

**DOI:** 10.1128/mbio.02375-22

**Published:** 2022-10-06

**Authors:** David da Silva Barreira, Pierre Lapaquette, Julia Novion Ducassou, Yohann Couté, Jean Guzzo, Aurélie Rieu

**Affiliations:** a University Bourgogne Franche-Comté (UBFC), Institut Agro Dijon, Dijon, France; b University Grenoble Alpes, INSERM, CEA, UMR BioSanté U1292, CNRS, CEA, FR2048, Grenoble, France; Universidade de Sao Paulo

**Keywords:** *Lacticaseibacillus casei*, membrane vesicles, membrane vesicle production, prophages, spontaneous prophage induction

## Abstract

The formation of membrane vesicles (MVs) by Gram-positive bacteria has gained increasing attention over the last decade. Recently, models of vesicle formation have been proposed and involve the digestion of the cell wall by prophage-encoded or stress-induced peptidoglycan (PG) hydrolases and the inhibition of PG synthesis by β-lactam antibiotics. The impact of these mechanisms on vesicle formation is largely dependent on the strain and growth conditions. To date, no information on the production of vesicles by the lactobacilli family has been reported. Here, we aimed to characterize the MVs released by the Gram-positive bacteria *Lacticaseibacillus casei* BL23 and also investigated the mechanisms involved in vesicle formation. Using electron microscopy, we established that the size of the majority of *L. casei* BL23 vesicles ranged from 50 to 100 nm. Furthermore, we showed that the vesicles were released consistently throughout the growth of the bacteria in standard culture conditions. The protein composition of the vesicles released in the supernatant was identified and a significant number of prophage proteins was detected. Moreover, using a mutant strain harboring a defective PLE2 prophage, we were able to show that the spontaneous and mitomycin-triggered induction of the prophage PLE2 contribute to the production of MVs by *L. casei* BL23. Finally, we also demonstrated the influence of prophages on the membrane integrity of bacteria. Overall, our results suggest a key role of the prophage PLE2 in the production of MVs by *L. casei* BL23 in the absence or presence of genotoxic stress.

## INTRODUCTION

Membrane vesicles (MVs) are spherical nanostructures enclosed by a lipid bilayer ranging from 20 nm to 400 nm in diameter. They were found to be produced by organisms of all three domains of life, namely, *Archaea*, *Bacteria*, and *Eukarya*, and can carry a diversity of components (1, 2). The composition of MVs is influenced by the organism that produces them and its physiological state, the mechanism of biogenesis, and environmental factors (1, 3, 4).

The release of bacterial MVs was first observed as early as 1966 in the Gram-negative bacteria Escherichia coli (5). Since then, research has mainly focused on studying Gram-negative vesicles as the production of vesicles by Gram-positive bacteria was considered unintuitive due to the presence of a thick peptidoglycan (PG) wall covering the bacteria. It took more than 20 years for the first clear mention of Gram-positive vesicles to appear in the literature (6, 7), and it is only recently that the production of vesicles by Gram-positive bacteria has received greater attention. The vesicles of Gram-positive bacteria have been found to carry a wide variety of cellular compounds, including proteins, lipids, and nucleic acids (8). Moreover, various roles have also been reported such as horizontal gene transfer, cell communication, antibiotic resistance, and virulence (3, 9).

To date, a limited number of studies have investigated the mechanism of MV biogenesis by Gram-positive bacteria (10–12). A recent study in Bacillus subtilis has shown that in treatment with a genotoxic agent (mitomycine C; MMC), the induction of a prophage-encoded holin-endolysin system triggered the formation of MVs through the digestion of the PG (12). Indeed, upon induction, the insertion of holins in the cell membrane allow endolysins to access the PG of the bacteria, while the hydrolase activity of endolysins create perforations in the bacterial wall. Thus, it is thought that the vesicles are formed by the extrusion of cytoplasmic membranes due to turgor pressure. In contrast to Gram-negative bacteria, the formation of vesicles does not lead to explosive cell lysis (13–15); instead, bacteria with impaired membrane integrity maintain their morphology while producing vesicles, a phenomenon called “bubbling cell death.” Similarly, another study has recently reported the role of prophage induction in vesicle release in MMC treatment in Lactococcus lactis (11). Several prophage-independent mechanisms of vesicle production have also been described in Gram-positive bacteria, including the inhibition of PG synthesis by β-lactam antibiotics (7, 16) and the digestion of the PG by the hydrolase activity of autolysins (17, 18).

Several publications have shown that bacteria from the lactobacilli family produce membrane vesicles and different functions have been attributed to them, including antimicrobial activities (19–22), cell communication, and immunomodulation (23–29).

Lactobacilli are Gram-positive bacteria widely used in industrial food fermentation processes and are “generally recognized as safe” (GRAS). Some strains of lactobacilli are part of the intestinal microbiota and may have probiotic effects. The World Health Organization (WHO) has defined probiotics as “live microorganisms which, when administered in adequate amounts confer a health benefit on the host” (30). Several probiotics have been demonstrated to enhance gastrointestinal health, in part by stimulating host immunity and inhibiting pathogen adherence to mucus and to intestinal epithelial cells (31, 32). Among probiotics, the dairy strain BL23 of the species *Lacticasibacillus casei* (formerly known as Lactobacillus casei) is widely studied due to its anti-inflammatory effects (33), its ability to prevent experimental colitis in mouse models (34), and its role in host defense against pathogens (35). Recently, *L. casei* BL23 was found to release MVs (36) but the mechanisms involved in vesicle biogenesis remain unclear. Thus, we aimed to investigate the mechanisms involved in the production of MVs by *L. casei* BL23.

In the present study, we report the characterization of the MVs released by *L. casei* BL23 in standard growth conditions using electron microscopy and MS-based proteomic analysis. We also quantify the amount of MVs released in the supernatant during bacterial growth. Furthermore, we demonstrate the contribution of spontaneous and mitomycin-triggered prophage induction in the production of MVs by *L. casei* BL23. Finally, we provide evidence of interactions between several prophages in *L. casei* BL23.

## RESULTS

### *L. casei* BL23 releases MVs during the bacterial growth.

**FIG 1:**
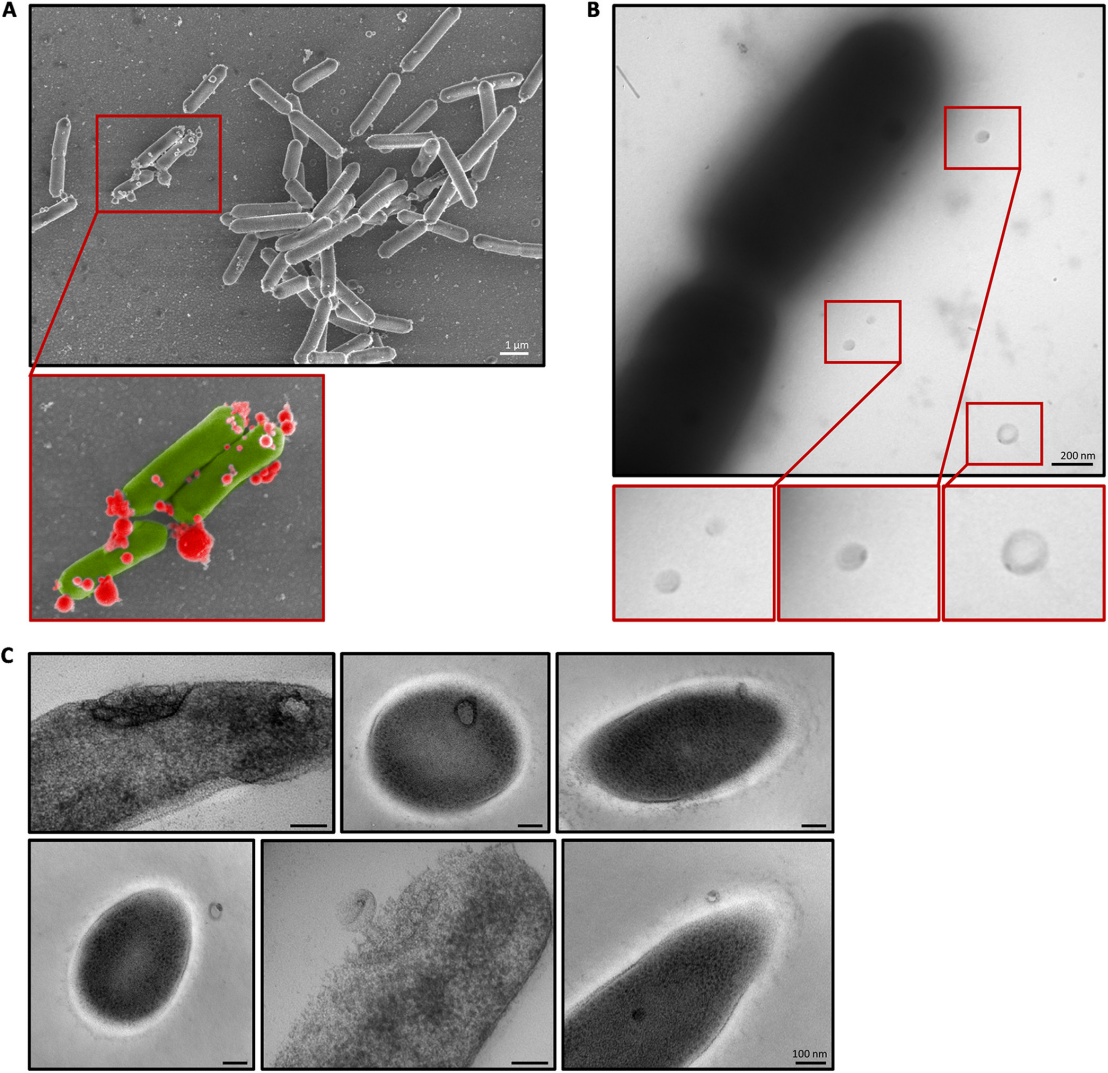
MVs are observed inside, on the surface and in the vicinity of *L. casei* BL23. (A) SEM image of MVs associated with the surface of the bacteria. In the lower image, MVs are colorized in red and the bacteria in green. (B) Negative-staining TEM images of MVs observed in the immediate vicinity of the bacteria. MVs are shown in the bottom panel. (C) HPF-FS TEM images of MVs closely associated with the bacteria.

The presence of MVs in the culture medium of *L. casei* BL23 was first investigated using several electron microscopy methods (namely, scanning electron microscopy [SEM], negative-staining, and high-pressure freezing [HPF-FS] transmission electron microscopy [TEM]). Using SEM, spherical structures were observed in the medium and associated with the surface of bacteria after 24 h of culture (Fig. 1A). For easier identification and differentiation, these structures were colored in red and bacteria were colored in green in the zoomed-in view (Fig. 1A). MVs were also observed next to the bacteria by negative-staining TEM (Fig. 1B). Surprisingly, all the MVs presented a more contrasted spot at one pole. Using HPF-FS TEM, we also observed vesicles inside the cytoplasm, associated with the cytoplasmic membrane, the cell wall, and in the culture medium outside the cells (Fig. 1C). Interestingly, some bacteria presented clusters of vesicles embedded in the bacterial envelope (Fig. 1C). Overall, these results suggest *L. casei* BL23 produce MVs associated with the bacterial envelope and released into the culture medium. It is noteworthy that only a small proportion of bacteria were found to be associated with vesicles, suggesting that in standard growth conditions (i.e., MRS medium at 37°C) few bacteria produce MVs.

**FIG 2:**
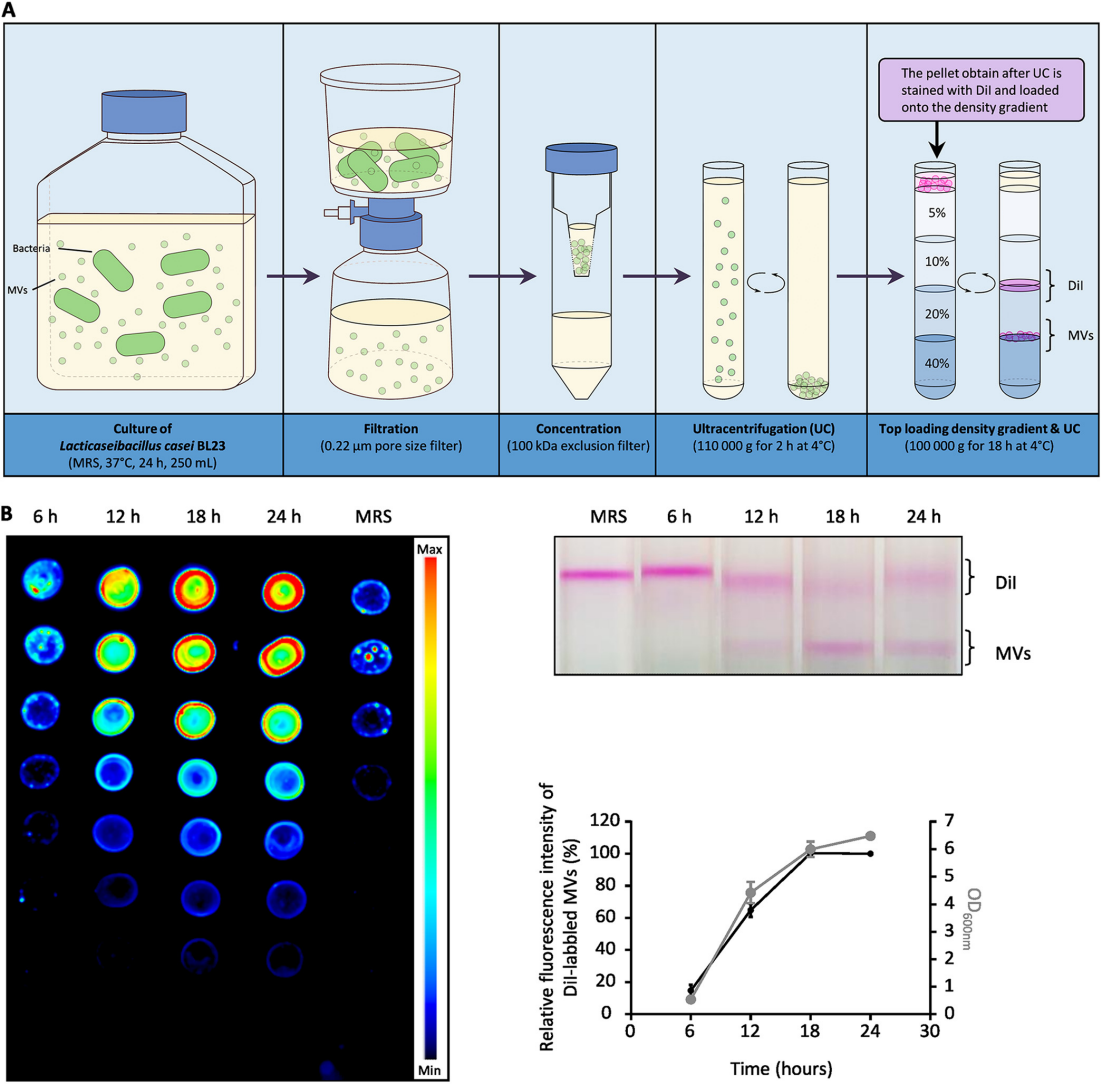
Most vesicles are released during the growth of the bacteria within the first 24 h of culture. (A) Schematic representation of the MV quantification protocol. After several steps of centrifugation, filtration, concentration, and ultracentrifugation, the MVs are stained with DiI before being loaded on the iodixanol density gradient. (B) After 6, 12, 18 and 24 h of growth, the amount of purified DiI-labeled MVs collected from the growth medium were compared. The left and the top right images show the fluorescence intensity of the DiI-labeled MV fractions collected at each time point and the corresponding gradients, respectively. The graph shows for each time point the relative amount of fluorescence emitted by the DiI-labeled MVs collected and the OD_600nm_ of the bacterial culture.

Next, we aimed to determine the kinetics of MV production during the first 24 h of bacterial growth. To achieve this, the MV quantification protocol described in Fig. 2A was used to quantify the vesicles released in the supernatant at 6 h, 12 h, 18 h, and 24 h of growth. This method is based on the purification of DiI-labeled vesicles by density gradient ultracentrifugation and the quantification of fluorescence intensity emitted by the vesicles. The quantification relies on the linear relationship between the amount of labeled vesicles and the intensity of fluorescence emitted (Fig. S1). We found that the quantity of DiI-labeled MVs increased mainly between 6 h and 18 h of growth (Fig. 2B). Conversely, between 18 h and 24 h, the amount of vesicles in the supernatant no longer increased (Fig. 2B). Moreover, we noticed a clear parallel between the growth of *L. casei* BL23 (OD_600nm_) and the quantity of MVs in the supernatant (Fig. 2B). These observations suggest that MVs were released consistently throughout the growth of the bacteria with the amount MVs released reflecting the amount of cell in the medium.

10.1128/mbio.02375-22.1 FIG S1 Validation of the relative quantification method of purified and DiI labeled MVs. After the first UC, several dilutions of the suspended pellet were prepared (q.s. 1 mL of PBS). The diluted solutions were then labeled with DiI 30 min at 37°C and loaded on top of the density gradient before the last UC, as described in the diagram (Fig. 2A). The fractions containing the DiI-labeled MVs were then carefully collected and used to prepare a serial dilution series. 5 μL of each dilution was then dropped into a nitrocellulose membrane (0.2 μm), dried at room temperature, incubated for 5 min in Tris-buffered saline (1X) and quantified using the Odyssey Fc imager (LI-COR). Download FIG S1, TIF file, 0.6 MB. Copyright © 2022 da Silva Barreira et al. 2022 da Silva Barreira et al. https://creativecommons.org/licenses/by/4.0/ This content is distributed under the terms of the Creative Commons Attribution 4.0 International license.

### The size and protein composition of the MV produced by *L. casei* BL23 were characterized by electron microscopy and proteomics.

To analyze the MVs released by *L. casei* BL23 into the supernatant, the vesicles were concentrated and purified after density gradient ultracentrifugation. The purified vesicles were then observed by electron microscopy (cryo-SEM, negative-staining TEM).

**FIG 3:**
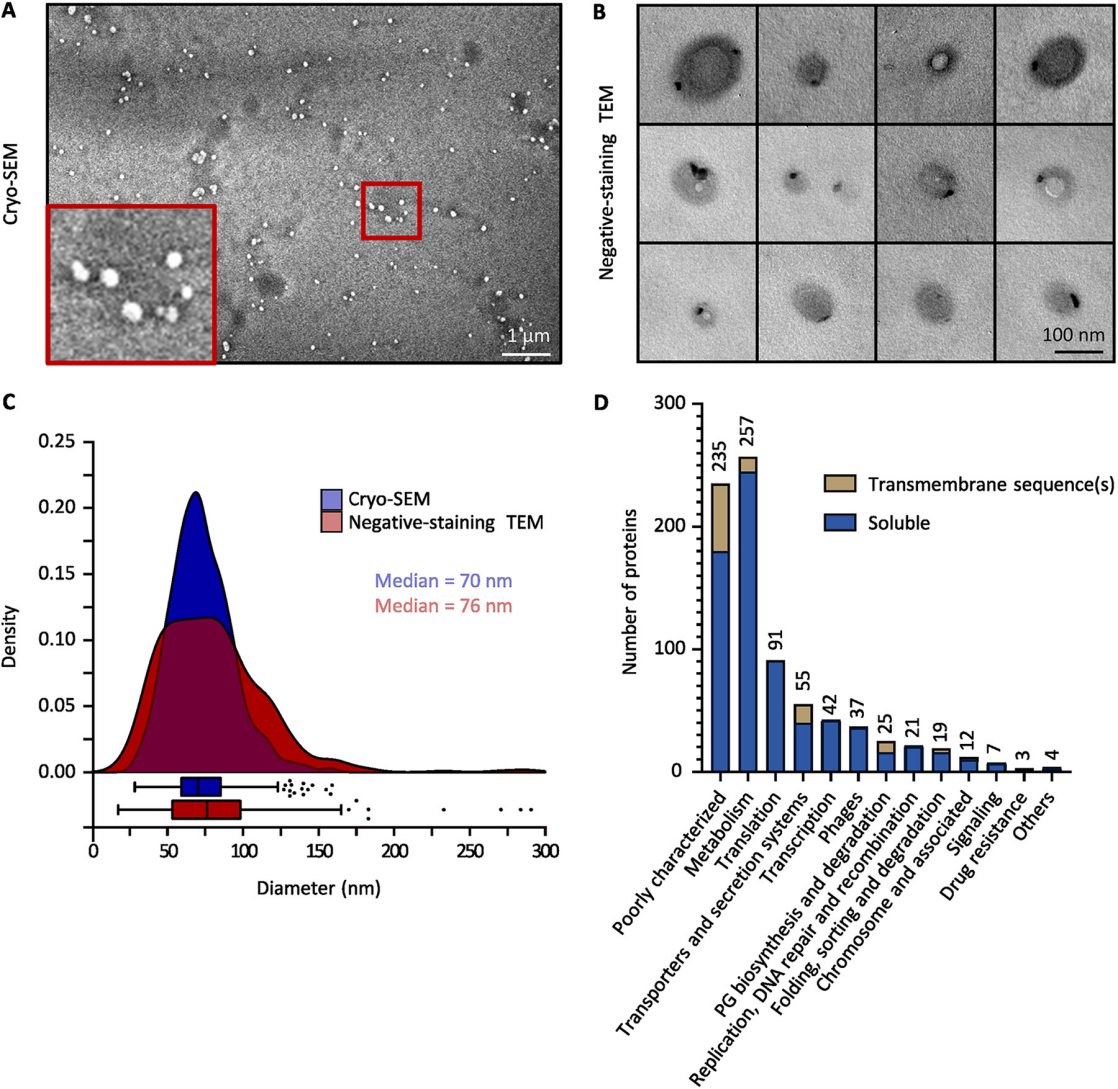
Purified MVs from *L. casei* BL23 are characterized for size and protein composition. (A) Cryo-SEM image of purified MVs. (A, inset) Zoomed-in view of the Cryo-SEM image. (B) Negative-staining TEM images of purified MVs. (C) The size distribution of purified MVs was obtained by analysis of the Cryo-SEM (830 vesicles) and the negative-staining TEM (533 vesicles) images. Vesicle sizes were obtained using the image analysis software Fiji (37). (D) Functional classification of *L. casei* BL23 MV proteins.

Using cryo-SEM, we observed spherical structures in the sample containing purified MVs (Fig. 3A) while no structures were found in the negative control (Fig. S2). The negative control corresponds to the fraction collected after carrying out the purification protocol on the culture medium alone (i.e., MRS medium) (Fig. S2). Consistent with our previous negative-staining TEM observations (Fig. 1B), we noticed that purified vesicles were also found to be polarized with a contrasted spot at one pole (Fig. 3B). Importantly, no phage heads, tails, or full phage particles were observed in the purified MV samples while analyzing negative-staining TEM images. The cryo-SEM images and the negative-staining TEM images were further analyzed with the Fiji software (37) to establish the size distribution of the purified MVs (Fig. 3C). With both microscopy methods, we saw that the size of MVs followed a normal distribution with the majority of the vesicles ranging from 50 nm to 100 nm (Fig. 3C). These results demonstrated that the purification protocol used allowed obtaining purified vesicles which were similar to the structures previously observed near the bacteria (Fig. 1B and C).

10.1128/mbio.02375-22.2 FIG S2 Cryo-SEM image of purified MVs. The image on the right (negative control) shows that no MVs were observed after the purification protocol was performed on the culture medium. Download FIG S2, TIF file, 1.2 MB. Copyright © 2022 da Silva Barreira et al. 2022 da Silva Barreira et al. https://creativecommons.org/licenses/by/4.0/ This content is distributed under the terms of the Creative Commons Attribution 4.0 International license.

To further explore the MVs produced by *L. casei* BL23, we then decided to examine the protein composition of the purified vesicles. MS-based proteomic analyses were performed on them and the proteins detected are listed in the Table S1. The functional classification of the proteins detected was obtained using the Kegg (38) and Uniprot (39) databases (Fig. 3D). *L. casei* BL23 vesicles contain a majority of soluble proteins, including several metabolic enzymes as well as translation and transcription associated proteins (Fig. 3D). In addition, it is interesting to note the presence of many proteins associated with the chromosome in the MV fraction, such as DNA replication, DNA repair, and DNA recombination proteins (Fig. 3D). We also detected a number of proteins associated with PG biosynthesis, protein folding, protein sorting, and protein degradation (Fig. 3D). Using the Phobius software (40), the presence of transmembrane sequences was predicted in 13% of all the proteins detected in the vesicular fraction. Noticeably, 37 proteins encoded by prophage sequences were identified in the vesicular fraction (Fig. 3D).

10.1128/mbio.02375-22.7 TABLE S1 MS-based proteomic analysis of three independent preparations of purified membrane vesicles from *Lacticaseibacillus casei* BL23. Download Table S1, XLSX file, 0.08 MB. Copyright © 2022 da Silva Barreira et al. 2022 da Silva Barreira et al. https://creativecommons.org/licenses/by/4.0/ This content is distributed under the terms of the Creative Commons Attribution 4.0 International license.

### The prophage PLE2 replicates its DNA and express proteins during the growth of *L. casei* BL23.

**FIG 4:**
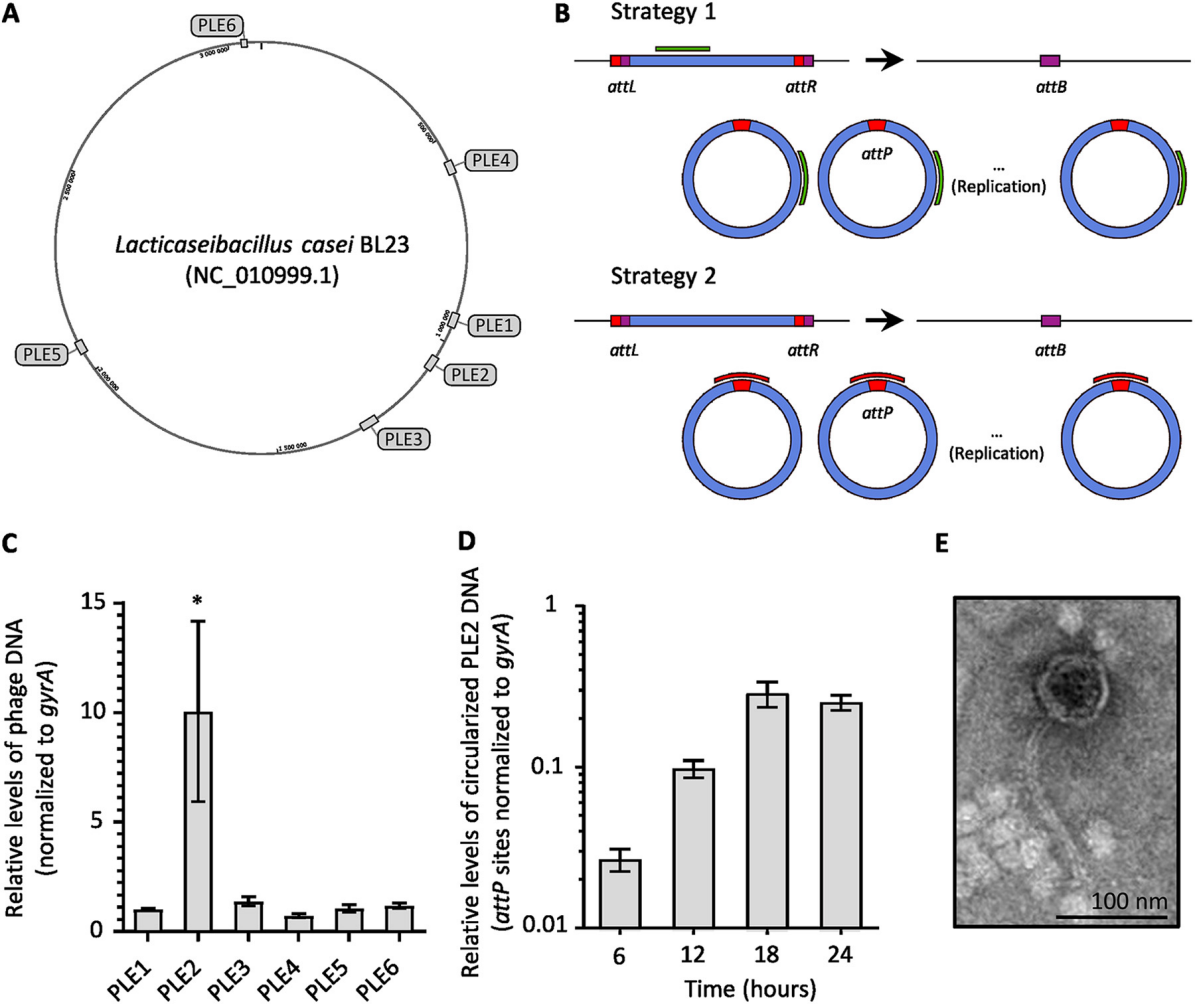
One out of the six predicted prophages in *L. casei* BL23 genome was able to replicate during the first 24 h of growth. (A) The position of six prophages in *L. casei* BL23 genome were predicted using the Phaster software. (B) Schematic drawings of the two strategies used to quantify prophage replication. Upon entry into the lytic pathway, the phage genome is replicated and the prophage excision generates *att*B/*att*P sites. Either a sequence within the prophage genome (green; strategy 1) or the *att*P sequence (red; strategy 2) is amplified and compared using qPCR with a bacterial genome sequence (*gyrA*). The bacterial (*att*B), phage (*att*P), and prophage (*att*L and *att*R) attachment-site sequences are indicated with colored boxes. (C) Relative quantification of the six putative phage genomes after 24 h of culture, using the qPCR strategy 1. (D) Quantification of the circularized PLE2 genome during the first 24 h of culture, using the qPCR strategy 2. (E) Negative-staining TEM images of purified PLE2 particles. After induction by MMC (400 ng/mL) at exponential growth phase (OD_600nm_ = 0.3), cells were incubated for 24 h before purification of phage particles by CsCl density gradient.

The identification of phage proteins in the vesicular fraction of *L. casei* BL23 suggests a possible role of prophages in the biogenesis of the MVs. To examine the role of prophages, we used the Phaster software (41) to predict the presence of prophage sequences in the chromosome of *L. casei* BL23. Six putative prophages were detected. The prophages PLE1 to PLE4 were described previously by Dieterle et al. (42). Our analysis predicted two more prophage sequences that we named PLE5 and PLE6 (Fig. 4A; Table 1). Surprisingly, proteins encoded by all the predicted prophage sequences were detected in the vesicular fraction. However, the majority of the phage proteins were encoded by the prophages PLE1, PLE2, and PLE3 with 7, 10, and 11 proteins detected, respectively (Fig. S3A and B).

**TABLE 1:** Predicted prophage sequences in the chromosome of *L. casei* BL23 (NC_010999.1) using the Phaster software

Region	Name	Region length (Kb)	Region position
1	PLE4	31.5	557,260 to 588,824
2	PLE1	34.1	928,671 to 962,821
3	PLE2	35.8	1,042,493 to 1,078,311
4	PLE3	46	1,248,493 to 1,294,544
5	PLE5	30.7	2,047,882 to 2,078,675
6	PLE6	13.6	3,031,592 to 3,045,245

10.1128/mbio.02375-22.3 FIG S3 Proteins from 6 putative prophages were identified in the MV fraction of *L. casei* BL23 by MS-based proteomics. (A) Pie chart showing the proportion of protein encoded by each predicted prophage sequences. (B) Alignment of the 6 predicted prophage sequences using the ProgressiveMauve software (90). The colored collinear blocks represent homologous DNA regions internally free from genomic rearrangements. The similarity profile contained in these collinear blocks indicates the average level of conservation between the corresponding DNA regions. The prophage genes are shown below each comparison as black outlined boxes on the plus (above horizontal line) and minus (below horizontal line) strands. Bleu boxes represent the proteins detected by proteomics in the MV fraction of *L. casei* BL23. The position of the integrated plasmid (pRV300) in the genome of the strain DDB001 is also indicated by a red triangle (genomic location: from 1 058 183 to 1 062 180 bp). Please note that pRV300 was inserted in the gene *lcabl_10980* (putative DNA primase) of the prophage PLE2. Download FIG S3, TIF file, 0.7 MB. Copyright © 2022 da Silva Barreira et al. 2022 da Silva Barreira et al. https://creativecommons.org/licenses/by/4.0/ This content is distributed under the terms of the Creative Commons Attribution 4.0 International license.

To determine whether the predicted prophages were able to replicate during the 24 h of culture, quantitative PCR (qPCR) were performed using primers designed to amplify a small prophage sequence as depicted in Fig. 4B. For each prophage, the amplified sequence is found in both the integrated form of the phage (prophage) and in its circularized form (strategy 1; Fig. 4B). This strategy was used because the phage attachment-sites (*att*P) were not known for all six putative prophages. The qPCR results showed that after 24 h of culture, there was a 10-fold increase in the PLE2 sequences compared with the bacterial reference sequences *gyrA* while no significant difference was observed for the other putative prophages. These results suggest the prophage PLE2 is the main prophage to replicate its genome during the first 24 h of growth (Fig. 4C). To further study the replication of PLE2, we used qPCR to amplify the *att*P site of PLE2 at four different time points during the growth of *L. casei* BL23 (strategy 2; Fig. 4B). Phage PLE2 replicated its DNA mostly before the first 18 h of bacterial growth (Fig. 4D). After 18 h, the quantity of PLE2 DNA did not increase (Fig. 4D). We also noticed the dynamics of phage replication was similar to the bacterial growth curve and the release of MVs (graph in Fig. 2B). This result suggests a relationship between phage replication and the release of MVs. Finally, we wondered if prophage PLE2 was able to produce complete phage particles. To answer this question, we treated *L. casei* BL23 with a DNA damaging agent (mitomycin C [MMC]) in the early exponential phase (OD_600nm_ = 0.3 to 0.4) and looked for phage particles after purification by CsCl density gradient using negative-staining TEM. As previously described by Dieterle et al. (42), the phage particles observed had the morphological characteristics of the *Siphoviridae* family (Fig. 4E) (43). The virions had long thin tails which seemed flexible, and icosahedral heads of about 70 nm.

### A *L. casei* BL23 strain harboring a deficient PLE2 prophage was obtained by insertional mutagenesis.

**FIG 5:**
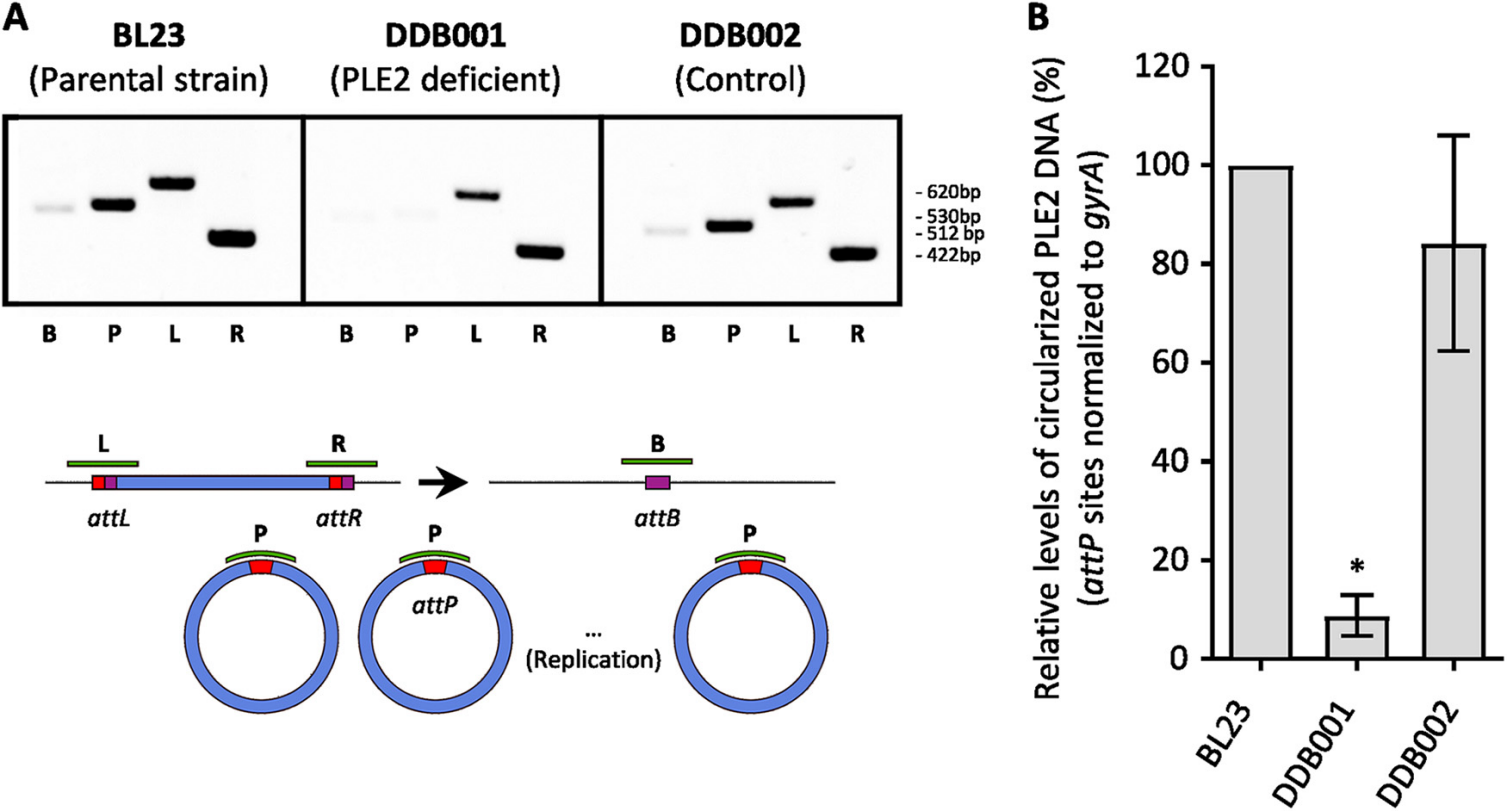
Construction of a *L. casei* BL23 strain harboring a PLE2 excision-deficient mutant. The replication of the phage PLE2 in the parental strain *L. casei* (BL23) is compared with the PLE2 deficient strain (DDB001) and with the control strain (DDB002) using PCR (A) and qPCR (B). The DDB001 strain was obtained by insertion of the pRV300 plasmid into the PLE2 prophage gene *LCABL_10980* (encoding a DNA primase). The DDB002 control stain was obtained by insertion of the pRV300 plasmid into a noncoding region. (A) For each strain, all attachment-site sequences were amplified by PCR and analyzed in a 1% agarose gel. (B) Comparison of the levels of circularized PLE2 in the parental, DBB001 and DDB002 strains using strategy 2 presented in Fig. 4B.

Based on previous publications establishing a link between prophage induction and vesicle release (10–12), we wanted to test whether the prophage PLE2 could be involved in vesicle production during the growth of *L. casei* BL23. To this end, we constructed a strain named DDB001 carrying a mutant of prophage PLE2. The mutant was obtained by integration of a nonreplicative plasmid into the prophage gene *lcabl_10980*, encoding a DNA primase (position of the plasmid is indicated by a red triangle in Fig. S3B). A control strain named DDB002 was also obtained by the insertion of the plasmid into an intergenic region of *L. casei* BL23 (Table S2). The plasmid insertion had no significant effect on the growth of strains DDB001 and DDB002 compared with the parental strain (Fig. S4A). In order to investigate the effect of mutagenesis on PLE2 replication, we used PCR to amplify the attachment sites *att*B, *att*P, *att*L, *att*R (named, respectively, B, P, L, and R) as described in Fig. 5A. Sites *att*L and *att*R can only be amplified by PCR if PLE2 is integrated in the chromosome of *L. casei* BL23. Conversely, the site *att*B is amplified only after the excision of the prophage while site *att*P is amplified after both the excision and circularization of the prophage genome (Fig. 5A). The PCR profiles obtained for the parental strains BL23 and the control strain DDB002 are similar. Bands corresponding to the amplification of the four attachment sites were observed, indicating that, within the bacterial population, both integrated and circularized forms of PLE2 were present. In contrast, for the DDB001 strain (PLE2 deficient), only the *att*L and *att*R amplicons were observed indicating that the mutation prevents excision of the PLE2 prophage (Fig. 5A). qPCRs were also performed to compare the levels of circularized PLE2 genome between the three strains (Fig. 5B). The *att*P site of PLE2 was amplified with the primers described in strategy 2 of the Fig. 4B. Consistently, we saw a significant decrease of the *att*P site in the DDB001 strain compared with the parental and the control strain (Fig. 5B). Finally, we examined the impact of mutagenesis on the replication of the other putative prophages by qPCR and no impact was observed in the standard growth condition (Fig. S4B).

10.1128/mbio.02375-22.4 FIG S4 The mutation in the prophage PLE2 neither affect the growth of the bacteria nor the replication of other putative prophages. (A) Growth curve showing the OD600nm of the parental, DDB001 and DDB002 strains during the first 24 hours of culture. (B) The replication of each putative prophage in the parental strain BL23, and in the DDD001 and DDB002 strains was quantified by qPCR, using the strategy 1 presented in Figure 4B. Download FIG S4, TIF file, 0.3 MB. Copyright © 2022 da Silva Barreira et al. 2022 da Silva Barreira et al. https://creativecommons.org/licenses/by/4.0/ This content is distributed under the terms of the Creative Commons Attribution 4.0 International license.

10.1128/mbio.02375-22.8 TABLE S2 Strains, plasmids, and oligonucleotides used in this study. Download Table S2, DOCX file, 0.02 MB. Copyright © 2022 da Silva Barreira et al. 2022 da Silva Barreira et al. https://creativecommons.org/licenses/by/4.0/ This content is distributed under the terms of the Creative Commons Attribution 4.0 International license.

### The replication of the PLE2 prophage leads to the production MVs by *L. casei* BL23.

**FIG 6:**
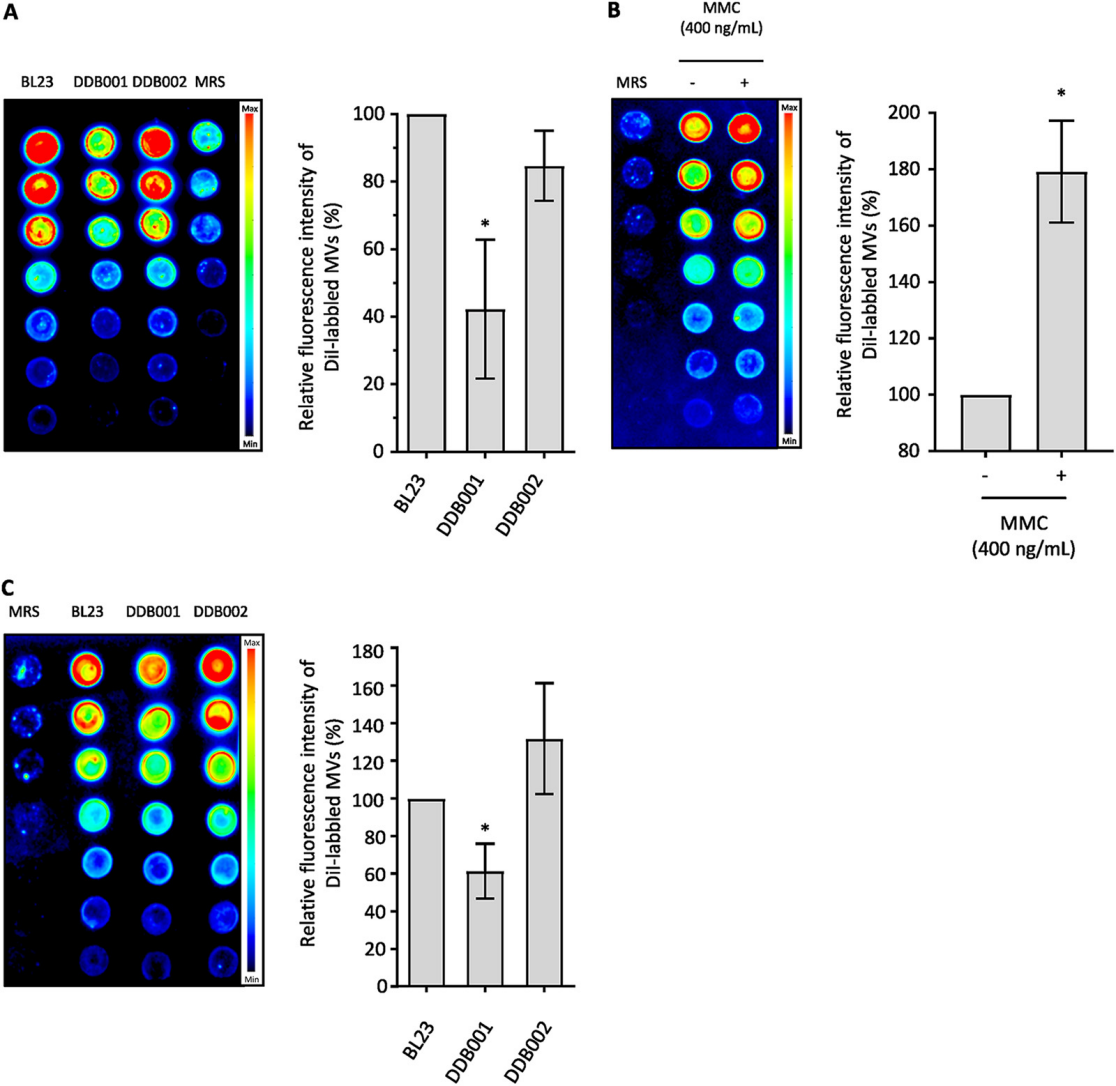
Contribution of the PLE2 prophage in the production of MVs with or without genotoxic stress. Relative quantification of the DiI-labeled MV fractions collected (A) from BL23, DDB001 and DDB002 strains after 24 h of culture or (B) from the BL23 strain treated with or without MMC. (C) Relative quantification of the DiI-labeled MVs collected from BL23, DDB001, and DDB002 strains treated with MMC. A final concentration of 400 ng/mL of MMC was added to the medium in the exponential phase (OD_600nm_ = 0.3 to 0.4) and the MVs were collected after 24 h of culture. The left image and the right graph show, respectively, a representative nitrocellulose membrane and the quantification of fluorescence emitted by the DiI-labeled MVs purified from each strain.

To investigate the contribution of the prophage PLE2 in MV production, we compared the quantity of MVs released by the strain DDB001 (harboring a deficient PLE2 prophage) to the parental strain and the control strain DBB002 (Fig. 6A). In order to achieve this, the MVs released in the supernatant were purified and labeled for quantification after 24 h of culture as previously described (Fig. 2A). We measured a 2-fold decrease in fluorescence from the MV fraction of the strain DDB001 compared with the fractions collected from the parental and DDB002 strains (Fig. 6A). This result showed the mutant DDB001 released fewer MVs than the parental and control strains, indicating that in the absence of PLE2 prophage replication, fewer MVs were released by *L. casei* BL23.

In order to confirm the previous observations, we decided to induce the entry of the prophage into the lysogenic pathway and compare the quantity of MVs released by *L. casei* BL23 with or without induction. Treatment with DNA-damaging agents such as UV light and MMC are commonly used in the literature to induce the switch of prophages to the lytic pathway. We chose to treat the bacteria in the early exponential phase (OD_600nm_ = 0.3 to 0.4) with 400 ng/mL of MMC. After 24 h of culture with or without MMC, the levels of prophage replication and the level of MVs released in the supernatant by *L. casei* BL23 were quantified. Compared with the nontreated condition, twice as much fluorescence was emitted by the MV fraction collected from the bacteria treated with MMC (Fig. 6B). In addition, we noted the production of MVs was also affected after MMC treatment in the mutant strain DDB001 compared with the parental and the control strains (Fig. 6C). qPCRs were performed as described previously (strategy 1; Fig. 4B) to examine the impact of MMC treatment on the replication of all the prophages (Fig. S5). As expected, MMC treatment induced an increase in the replication of the prophage PLE2. In addition, we also noticed a slight induction of prophages PLE1 and PLE3 in the treated condition. No significant impact was observed on the replication of other phages (Fig. S5).

10.1128/mbio.02375-22.5 FIG S5 Treatment with MMC affects the replication of PLE2. The effect of the MMC treatment on the replication of each putative prophage in the parental strain BL23 was quantified by qPCR, using strategy 1 presented in Figure 4B. Download FIG S5, TIF file, 0.1 MB. Copyright © 2022 da Silva Barreira et al. 2022 da Silva Barreira et al. https://creativecommons.org/licenses/by/4.0/ This content is distributed under the terms of the Creative Commons Attribution 4.0 International license.

Altogether, these results showed a strong relationship between the induction of PLE2 and the release of MVs, suggesting a contribution of PLE2 in the production of MV by *L. casei* BL23.

### The induction of the prophage PLE2 affects membrane integrity and promotes MV release.

Next, we wanted to investigate if the induction of the prophage PLE2 could impact the membrane integrity of the bacteria.

**FIG 7:**
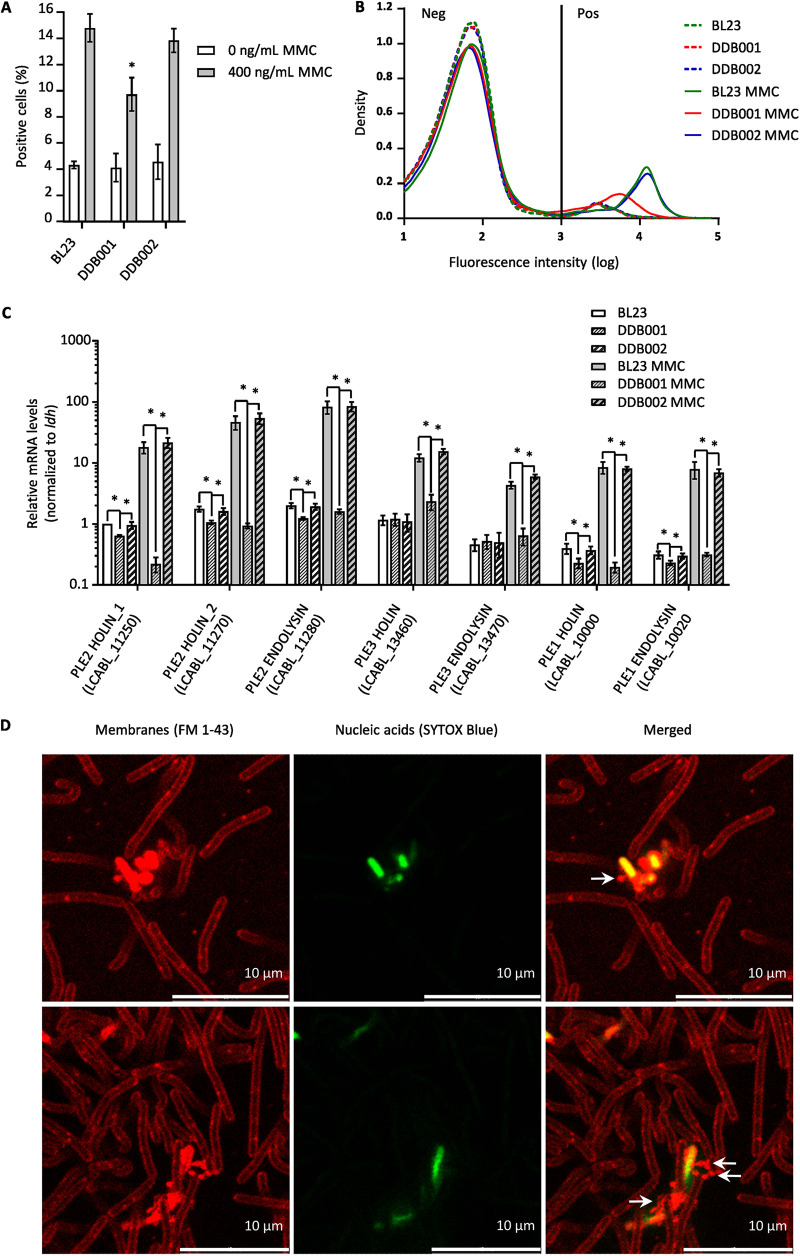
The PLE2 prophages affect the permeability of *L. casei* BL23 leading to the release of MVs. (A and B) BL23, DDB001, and DDB002 strains treated with or without MMC were analyzed by cytometry to quantify the proportion of permeable cells within each population. A final concentration of 400 ng/mL of MMC was added to the medium in the exponential phase (OD_600nm_ = 0.3 to 0.4) and after 24 h of culture; permeable cells were labeled with a membrane-impermeable nucleic acid stain (SYTOX blue). The proportion of positive cells (A) and the distribution of fluorescence intensity (B) are shown for each strain. A minimum of 4.10^4^ events per replicate were analyzed. (C) The relative expression of the putative holin-endolysin systems was measured by reverse transcription-qPCR (RT-qPCR) in the BL23, DDB001 and DDB002 strains treated with or without MMC (400 ng/mL). Holin-endolysin systems were detected only in prophages PLE1, PLE2, and PLE3. (D) Confocal microscopy images of *L. casei* BL23 cells. After 24 h of culture, cell membranes were labeled with the lipophilic dye FM 1–43 (red) and permeable cells were stained with SYTOX blue (green).

To measure the influence of prophage PLE2 on membrane integrity, we used flow cytometry to count the number of permeable cells in the population of the 3 strains (*L. casei* BL23, DDB001 and DDB002) treated with or without MMC (400 ng/mL). Permeable cells were stained with SYTOX Blue, a nucleic acid stain that penetrates cells with compromised cytoplasmic membranes (Fig. 7A and B). When treated with MMC, we observed a significant decrease in the number of positive cells in the DDB001 population compared with the parental and control populations, suggesting that there were fewer permeable bacteria in the population of the DDB001 strain (Fig. 7A). Furthermore, we also noticed that the fluorescence intensity of the DDB001 positive cells was lower than the other two strains, indicating that the positive DDB001 cells were less fluorescent and, therefore, less permeable (Fig. 7B). Remarkably, we also noticed that the MMC-treated cells were longer than the nontreated cells (Fig. S6A). In particular, the DDB001 treated cells were twice as long as the parental and control strains in the presence of MMC treatment and five times longer than the nontreated cells (Fig. S6A). Without MMC treatment, fewer positive cells were observed for all strains compared with the treated condition (Fig. 7A). In addition, no differences in cell permeability were observed between the different strains without MMC treatment (Fig. 7B and C). These results suggest that when cultured without stress, the number of permeable cells caused by the induction of PLE2 is too low to measure differences between strains.

10.1128/mbio.02375-22.6 FIG S6 Impact of the PLE2 mutation on bacterial morphology after MMC treatment and on the replication of other putative prophages. (A) The size distribution of BL23, DDB001 and DDB002 strains treated with or without MMC (400 ng/mL) were obtained by cytometry. (B) The effect of MMC treatment on the replication of each putative prophage was compared by qPCR in BL23, DDB001 and DDB002 strains, using strategy 1 presented in Figure 4B. Download FIG S6, TIF file, 0.4 MB. Copyright © 2022 da Silva Barreira et al. 2022 da Silva Barreira et al. https://creativecommons.org/licenses/by/4.0/ This content is distributed under the terms of the Creative Commons Attribution 4.0 International license.

Previous works on Gram-positive bacteria (11, 12) have shown that the disruption of the cytoplasmic membrane and the digestion of PG by the holin-endolysin systems of prophages are responsible for the production of MVs. The model proposes that the insertion of holins in the cytoplasmic membrane allows the PG hydrolases (endolysins) to access and digest the walls of the bacteria. Then, the perforation in the PG created by the endolysins leads to the budding of the membrane and the formation of vesicles.

In *L. casei* BL23 genome, holin-endolysin systems were predicted in prophages PLE1, PLE2, and PLE3 using the Phaster software. We would like to mention that two hypothetical holins referred to as holin_1 (*lcabl_11250*) and holin_2 (*lcabl_11270*) were predicted next to each other in the prophage PLE2. To clarify the contribution of each prophage on cell permeability, we quantified the expression of the predicted holin-endolysin systems in the three strains (BL23, DDB001, and DDB002) with or without MMC treatment. Interestingly, we observed that all the holin-endolysin systems were induced in the MMC-treated conditions (Fig. 7C). The holin (*lcabl_11270*) and the endolysin (*lcabl_11280*) of prophage PLE2 were the most expressed. Furthermore, no activation of the holin-endolysin genes were observed in the DDB001 strain with or without MMC treatment. These results show that none of the three predicted prophage holin-endolysin systems were expressed in the DDB001 strain, in accordance with the previous results showing a lower number of permeable cells in the bacterial population compared with the parental and control strains (Fig. 7A). These results suggest the mutation of the DNA primase (*lcabl_10980*) in DDB001 affects the entry into the lytic pathway of PLE2 which might explain why expression of genes required for the lytic development of the prophage are not induced.

To further investigate the contribution of each prophage, qPCRs were performed to determine their level of replication in the three strains (BL23, DDB001, and DDB002) after MMC treatment, as described previously (strategy 1; Fig. 4B). Consistent with our previous results (Fig. S5), a significant increase in the replication of prophage PLE2 was observed in the parental strain (BL23) and in the control strain (DDB002) compared with the nontreated condition (Fig. S6B). A slight increase in the PLE3 and PLE1 sequences was also observed in the treated condition for the parental and control strains as opposed to the DDB001 strain. (Fig. S6B). Furthermore, no induction of prophage PLE2 was observed in DDB001, with or without MMC treatment (Fig. S6B).

Altogether, the absence of PLE2 replication and the absence of holin endolysin expression explains the reduced number of permeable cells in the DDB001 strains. These observations are in agreement with the hypothesis that the induction of prophage PLE2 contributes to the formation of MVs through the alteration of membrane integrity and the digestion of PG.

To further support the results presented above, we finally wanted to investigate if permeable cells released MVs. In order to achieve this, bacteria were observed by confocal fluorescence microscopy after double staining (Fig. 7D). Permeable cells were labeled with SYTOX Blue and cell membranes were stained with the membrane probe FM 1–43. As expected, we noticed spherical membrane structures (red, indicated by white arrows) on the surface of permeable bacteria (green) (Fig. 7D). It is interesting to note that these observations are similar to the observation made using SEM (Fig. 1A). Indeed, in both the confocal microscopy images (Fig. 7D) and the SEM images (Fig. 1A), we can see bacterial cells covered with similar vesicular structures.

## DISCUSSION

The release of membrane vesicles by *L. casei* was first described in 1992 (7) and recent publications have also reported the production of MVs by this species (27, 36, 44, 45). Consistent with the literature, we showed by electron microscopy that *L. casei* BL23 produces MVs which were found in the cytoplasm, associated with the surface of the bacteria and free in the supernatant. We believe that the presence of intracellular vesicles could be due to the invagination of the cytoplasmic membrane, as described in Streptococcus *pneumonia* (46) and in Bacillus anthracis (47). Interestingly, we also observed that some bacteria presented accumulations of internal vesicles located near the wall, comparable with the structures observed in Bacillus anthracis (47). Furthermore, with TEM we noticed that almost all the MVs presented a contrasted spot at one pole. This polarization could be a staining artifact or the result of a specific characteristic of *L. casei* BL23 vesicles. However, as yet, we have no element to explain this observation.

Several publications have demonstrated that the production of MVs is closely linked to the growth phase of the bacteria (48–50). For *L. casei* BL23, we showed that in standard conditions (i.e., MRS medium at 37°C), MV production occurs throughout the growth of the bacteria during the first 24 h of culture. In accordance with the literature, the size of the vesicles ranged from 20 nm to 200 nm with an average of 75 nm (27, 36).

To determine the protein content of *L. casei* BL23 vesicles, MS-based proteomics were used to analyze the vesicular fraction obtain after density gradient purification. A total of 810 proteins were detected, including a majority of metabolic, translational, and transcriptional proteins. Moreover, the presence of chromosome-associated proteins in the vesicular fraction, such as DNA polymerases and transcription factors, is consistent with previous results showing that the vesicles of *L. casei* BL23 carry nucleic acids (36). In addition, Rubio et al. showed that some proteins of *L. casei* BL23 were particularly enriched in the vesicles compared to the whole-cell extracts (36). The literature indicates that the enrichment of proteins in the vesicles can be due to either a specific mechanism of vesicle formation allowing protein selection, or a significant contribution of a subpopulation expressing a specific set of proteins (4, 51). The presence of capsid proteins in the vesicular fraction of *L. casei* BL23 supports the idea that vesicles were produced by a subset of the bacterial population in which prophages switched from the lysogenic to the lytic pathway.

Despite the absence of MMC induction, we detected proteins encoded by all the prophage sequences of *L. casei* BL23 in the vesicular fraction. This observation might be explained by the fact that bacteria are known to express and use proteins from cryptic and functional prophages for their own benefit (52, 53). Prophages are also known to express some regulatory genes when they are in their latent form (54). Interestingly, we noticed that most of the proteins detected were encoded by PLE2, PLE3, and PLE1 (10, 11, and 7 proteins, respectively), which is consistent with the finding that they are the only complete prophages (42). The interaction between PLE2, PLE3, and PLE1 for gene expression and replication support the idea that certain proteins encoded by one prophage could also be used by other prophages; thus, explaining the presence of many prophage proteins in the MVs of *L. casei* BL23.

In accordance with the literature (55), we showed prophage PLE2 is spontaneously induced during bacterial growth in standard culture conditions. In the absence of genotoxic stress, spontaneous induction was evidenced by the replication of PLE2 in the early and midexponential phase. No significant replication of PLE2 was observed in the stationary phase. Moreover, we observed that PLE2 was the only prophage to replicate its DNA in standard growth conditions. Spontaneous prophage induction (SPI) is a phenomenon known to impact the fitness of bacterial populations by promoting biofilm formation, horizontal gene transfer (HGT), and virulence (56–58). Notably, SPI has been reported in several lactic acid bacteria (10, 56, 57). Our work demonstrated that in the PLE2 deficient strain (DDB001), the absence of prophage replication was associated with a reduction in vesicle production. This result suggests that the spontaneous induction of PLE2 contributes to the release of MVs during bacterial growth. Moreover, the similarity between the PLE2 replication curve and the amount of MV released further supports this hypothesis. SPI was previously found to promote vesicle production in Gram-negative bacteria (49). For the first time, we proved that SPI also contributes to the production of vesicles in a Gram-positive bacterium.

The impact of MMC-induced prophages in the production of MVs has recently been shown in the Gram-positive bacteria Bacillus subtilis (12), Staphylococcus aureus (16), and *Lactococcus* (10, 11). Upon treatment with a genotoxic agent, SOS gene activation in the bacteria triggers a switch of prophages to the lytic pathway. Activation of the SOS response also inhibits cell division and can be evidenced by significant cell filamentation (59–61). We observed that the cells of the mutant strain DDB001 were much longer than the parental and control strains in the presence of MMC. This increase in length can probably be explained by the absence of PLE2 induction in the DDB001 population. Consequently, we can assume that the absence of holin-endolysin expression in the DDB001 strain might explain the reduction in the amount of vesicles released compared with the parental and control strain.

In agreement with this observation, the MMC-triggered induction of PLE2 affected the membrane integrity of the parental and control strains, as evidenced by the increase in cell permeability. It has recently been shown that the expression of holin-endolysin systems is responsible for the alteration of membrane integrity in Gram-positive bacteria (11, 12). The PG hydrolase activity of endolysin creates “holes” in the cell wall of the bacteria which leads to the budding of vesicles. The bacteria eventually lose their membrane integrity and form what has been described as “ghost cells.” This mechanism of vesicle biogenesis has been called “bubbling cell death” (4, 12). Depending on the bacterial species, the perforations in the cell wall lead to budding or the formation of nanotubes (62). These species-dependent phenomena probably depend on turgor pressure as well as the thickness and diameter of the wall perforations.

We observed that without genotoxic stress, the cell permeability of the DDB001 strain was similar to that of the parental and control (DDB002) strains. Our interpretation is that the number of permeable cells due to the spontaneous induction of the PLE2 prophage is much lower than the cell permeability due to prophage independent mechanisms, such as cell death. This result is consistent with the data indicating that spontaneous switching to the lytic state of PLE2 is estimated to occur in less than 0.7% of *L. casei* BL23 cells grown in standard conditions (42).

In *L. casei* BL23, prophages PLE1, PLE2, and PLE3 possess holin-endolysin systems which are all expressed upon MMC treatment. However, the PLE2 system is the most expressed after induction, and only the PLE2 holin (*lcabl_11270*) was detected in the vesicular fraction in the absence of stress. It is noteworthy that the endolysin activity of genes *lcabl_11280* (PLE2) and *lcabl_10020* (PLE1) were previously characterized by Regulski et al. (63). Furthermore, no expression of the holin-endolysin system was detected upon MMC treatment in the mutant strain DDB001. Strikingly, we also noticed that in the DDB001 strain, the expression of the holin-endolysin systems of all three prophages was impaired, both in the absence or presence of genotoxic stress. Indeed, along with the PLE2 prophage, no induction of the holin-endolysin systems encoded by prophages PLE1 and PLE3 was observed in the presence of MMC treatment. Similarly, we observed that upon MMC treatment, prophages PLE3 and PLE1 were slightly induced in the parental strain. However, no replication of prophages PLE1 and PLE3 were observed in the DDB001 strain carrying a PLE2-deficient prophage. Taken together, these results prove that prophages PLE1 and PLE3 rely on prophage PLE2 for their replication. Both PLE3 and PLE1 prophages display a dependent behavior toward PLE2 comparable with satellite prophages, like the P4 bacteriophage (64, 65). Reciprocally, we suspect that PLE2 could also utilize the other prophage proteins to facilitate its lytic cycle. This idea is further supported by the presence of strongly similar DNA regions between PLE2 and PLE1 (Fig. S3B). Therefore, the mutual dependence that exists between *L. casei* BL23 prophages could explain why several phage proteins were found in the MV fraction. Based on our observations, we believe the alteration of membrane integrity upon MMC treatment primarily involves the induction of PLE2 and its lysin-endolysin system. In agreement with this hypothesis, we showed an increase in cell permeability coupled with an increase in vesicle release after PLE2 induction upon MMC treatment.

As proposed in the “bubbling cell death” model (4, 12), using confocal microscopy we observed that initially the release of vesicles by permeable “ghost cells” did not lead to morphological modifications of the bacteria. Indeed, both in confocal microscopy and TEM, we were able to observe bacteria releasing vesicles while maintaining their morphology. However, when the cells were severely damaged, they eventually exploded and lost their shape (12).

Interestingly, we saw the number of permeable cells in the DDB001 population with MMC was twice as high as the parental strain without MMC. This result reveals a reduction in membrane integrity independent of prophage induction in the DDB001 strain and suggests that MMC strongly impact the physiology of the bacteria. This impact was also evidence by the significant cell filamentation in the DDB001 strain compared with the parental and control strains. Other mechanisms such as activation of the SOS response could potentially induce the production of vesicles in a phage-independent manner (61), thus explaining the production of vesicles by the DDB001 strain.

Our results showed that the PLE2-deficient strain DDB001 releases vesicles when cultivated with or without genotoxic stress. We interpret this observation as an indication that phage-independent mechanisms might be involved in the biogenesis of these MVs. Prophage-independent mechanisms of biogenesis have been reported in several Gram-positive bacteria (16–18). Notably, the inhibition of PG synthesis by β-lactam antibiotics was described regarding their role in MV production in Staphylococcus aureus and Lactobacillus casei ATCC 7469 (7, 16), as well as in Gram-negative bacteria (66–68). In addition, the role of autolysins in the release of MVs has been proved in Bacillus subtilis and Staphylococcus aureus (17, 18). Autolysins are a type of PG hydrolases that digest the cell wall and can be potentially lethal for the producing bacteria (69). Remarkably, we found a large number of PG hydrolases in the vesicular fraction of *L. casei* BL23, among which were several autolysins (70). These enzymes could potentially explain how the DDB001 strain produces vesicles independently of prophage induction. The presence of autolysin in the vesicles has been reported in many Gram-positive bacteria, including Bacillus subtilis (17), Staphylococcus aureus (71), Mycobacterium tuberculosis (72), Listeria monocytogenes (73), and Streptococcus suis (74). Furthermore, it has been shown that autolysins carried by MVs promote vesicle production from adjacent bacteria through the degradation of their cell wall (22, 75, 76).

### Conclusion.

In conclusion, our work has shown that spontaneous and MMC-triggered prophage induction contributes to the production of MVs by *L. casei* BL23 during cell growth. We have also gained new insights into the complex interactions that exists between the different prophages carried by the chromosome of *L. casei* BL23. The dependency of both PLE3 and PLE1 on prophage PLE2 strongly suggests their satellite nature. Vesicles were shown to play a large number of physiological roles: they are involved in stress resistance, biofilm formation (15, 77, 78), HGT (6, 9, 79), antibiotic (66–68, 71), and phage resistance (80). In this context, it will be relevant to further investigate whether the vesicles released after spontaneous and triggered prophage induction promote bacterial resistance to stresses.

## MATERIALS AND METHODS

### Bacterial strains and growth conditions.

The bacterial strains and plasmids used in this study are listed in Table S2. Unless otherwise noted, *L. casei* strains were cultivated anaerobically in Man-Rogosa-Sharpe medium (MRS; Conda) at 37°C under static conditions for 24 h. *L. casei* mutants obtained by plasmid integration were selected in solid MRS (1.5% agar) and maintained in liquid MRS supplemented with 5 μg/mL of erythromycin. Escherichia coli DH5α was grown in LB medium at 37°C under agitation and was used as a host for cloning. E. coli transformants were selected on solid LB (1.5% agar) supplemented with 100 μg/mL of ampicillin.

### Construction of plasmids and mutant strains.

The construction of *L. casei* BL23 insertion mutants was adapted from Leloup et al. and Muñoz-Provencio et al. (81, 82). First, the internal fragment of the gene *LCABL_10980* (encoding a DNA primase) and a noncoding region (accession number NC_010999) were amplified by PCR (Q5 High-Fidelity DNA polymerase NEB) using the *L. casei* BL23 genome as a template. The primers used for the PCR amplification are listed in Table S2. The amplified fragments were then cloned into the integrative vector pRV300 digested with EcoRI and HindIII/SacI restriction enzymes. The resulting recombinant plasmids (pRV001 and pRV002) were transformed in *L. casei* BL23 by electroporation with a GenePulser (Bio-Rad), as described in Posno et al. (83). Transformant bacteria were selected on MRS plates supplemented with 5 μg/mL of erythromycin. The correct integration of the plasmids was validated by PCR and sanger sequencing. As a result, the DDB001 mutant strain was created by integrating the plasmid (pRV001) into gene *LCABL_10980*. Similarly, the DDB002 control strain was created by inserting the plasmid (pRV002) into the intergenic region of the *L. casei* BL23 genome, starting from base 2441051 to 2441492 (accession number NC_010999).

### Purification and quantification of MVs.

For MV purification, we used a protocol adapted from a previous study (84). *L. casei* culture (250 mL) was centrifuged at 4,000 × *g*, 4°C for 20 min. The supernatant was filtered through a 0.22-μm pore size filter (Nalgen Rapid-Flow) to remove remaining bacterial cells and concentrated by ultrafiltration at 4,000 × *g*, 4°C with 100 K Amicon Ultra-15 centrifugal filters (Merck Millipore). The concentrated supernatant was filtered through a 0.22-μm-pore-size filter before ultracentrifugation (UC) at 110,000 g, 4°C for 2 h. The UC pellet was resuspended in sterile PBS (1×) and loaded on top of an iodoxinol gradient with layers of 5%, 10%, 20%, 40% of iodoxinol (corresponding to about 1.054, 1.079, 1.127, 1.223 g/mL, respectively) and UC at 100,000 × *g* for 18 h at 4°C. The fraction containing the MVs was then collected and resuspended in PBS (1×) before a final UC at 110,000 × *g* for 2 h at 4°C. Please note that the presence or absence of MVs in the different fractions of the density gradient was checked using negative-staining TEM. MVs were only observed at the interface formed between the layers of 1.127 and 1.223 g/mL (corresponding to 20 and 40% of iodixanol).

To quantify the MVs, the first UC pellet containing MVs was stained for 30 min with 1% Vybrant DiI at 37°C. The DiI-labeled MVs were then loaded on top of an iodoxinol gradient of 5% to 40% and UC at 100,000 × *g* for 18 h at 4°C. After the UC, the DiI-labeled MVs were carefully collected and used to prepare several dilutions and 5 μL of each dilution was then dropped into a nitrocellulose membrane (0.2 μm; Bio-Rad). From top to bottom 100%, 75%, 50%, 25%, 12.5%, 6.25%, 3.125% of each sample were spotted on the nitrocellulose membrane (0.2 μm; Bio-Rad), dried at room temperature, and then washed for 5 min in Tris-buffered saline (1×). The fluorescence emitted by the DiI-labeled MVs was quantified using the Odyssey Fc imager (LI-COR). The background level emitted by the spots of the negative control was subtracted from the fluorescence intensity of each sample according to their dilution. The negative control corresponds to the fraction collected after carrying out the purification protocol on the culture medium alone (i.e., MRS medium).

### Mass spectrometry-based proteomic analyses.

Three independent preparations of extracellular vesicles were analyzed. Proteins were solubilized in Laemmli buffer and heated for 10 min at 95°C. They were then separated by SDS-PAGE (4% to 12% NuPAGE, Life Technologies) and stained with Coomassie blue R-250 (Bio-Rad) before in-gel digestion using modified trypsin (Promega, sequencing grade) as described previously (85). For each replicate, the two very intense bands were prepared separately from the rest of the sample. The resulting peptides were analyzed by online nanoliquid chromatography coupled to MS/MS (Ultimate 3000 RSLCnano and Orbitrap Exploris 480 for the second experiment, Thermo Fisher Scientific) using a 35-min gradient for intense bands and a 120-min gradient for the rest of the samples. To do this, the peptides were sampled on a precolumn (300 μm × 5 mm PepMap C18, Thermo Fisher Scientific) and separated in a 75 μm × 250 mm C18 column (Reprosil-Pur 120 C18-AQ, 1.9 μm, Dr. Maisch). The MS and MS/MS data were acquired by Xcalibur (Thermo Fisher Scientific).

Peptides and proteins were identified by Mascot (version 2.7.0.1, Matrix Science) through concomitant searches against the Microscope database (86) (Lactobacillus casei BL23 taxonomy, April 2021 download), the Uniprot database (Saccharomyces cerevisiae S288c and Bos Taurus taxonomies, June 2021 download), and a homemade database containing the sequences of classical contaminant proteins found in proteomic analyses (keratins, trypsin, etc.). Trypsin/P was chosen as the enzyme and two missed cleavages were allowed. Precursor and fragment mass error tolerances were set at, respectively, 10 ppm and 20 ppm. Peptide modifications allowed during the search were: Carbamidomethyl (C, fixed), Acetyl (Protein N-term, variable), and Oxidation (M, variable). The Proline software (version 2.1) (87) was used for the compilation, grouping, and filtering of the results (conservation of rank 1 peptides, peptide length ≥ 6 amino acids, false discovery rate of peptide-spectrum-match identifications < 1% [88], and a minimum of one specific peptide per identified protein group). Proline was then used to perform the compilation, grouping and spectral counting-based comparison of the identified protein groups.

Proteins identified in the contaminant, bovine, and yeast databases were discarded from the final list of identified proteins.

### Transmission electron microscopy.

Negative staining was performed on bacterial suspension, on purified MVs and on purified phage suspensions before observation by TEM at the DimaCell platform (http://www.dimacell.fr/). Then, 10 μL of the sample was dropped on collodion-coated and carbon-stabilized nickel microscope grids and left for 3 min to allow the MVs, the bacteria, or the phages to bind. Excess liquid was gently blotted with Whatman paper and stained with 10 μL of 1% (wt/vol) uranyl acetate solution for 10 s. The grid was dried and observed using a Hitachi H7500 transmission electron microscope (Hitachi Scientific Instruments Co., Tokyo, Japan) operating at 80 kV and equipped with an AMT camera driven by AMT software (AMT, Danvers, MA, USA).

Bacteria were also observed after HPF-FS and freeze substitution at the DimaCell platform (http://www.dimacell.fr/). Bacterial cultures were centrifuged at 4,000 × *g* for 5 min and the pellets were cryoimmobilized using a Leica high-pressure freezer (Leica Microsystems, Vienna, Austria). Frozen samples were then freeze-substituted with a Leica EM AFS (Leica Microsystems, Vienna, Austria) in pure acetone containing 2% (wt/vol) osmium tetraoxide and 0.1% (wt/vol) uranyl acetate at −90°C for 72 h. The temperature was gradually increased (5°C/hour) to 4°C, kept at 4°C for 2 h and then finally kept for 1 h at room temperature. The samples were washed for 1 h in acetone at room temperature and infiltrated in increasing concentrations of Epon acetone mixtures until pure Epon-812 was obtained. Finally, the samples were embedded and polymerized in Epon-812 at 60°C for 48 h. Ultrathin sections (50 nm to 60 nm) were obtained with a ultramicrotome UC61 (Leica Microsystems, Vienna, Austria) and placed on collodion-coated and carbon-stabilized nickel microscope grids. The sections were stained with 1% (wt/vol) uranyl acetate for 30 min.

### Scanning electron microscopy.

Bacteria were fixed in 2.5% glutaraldehyde for 30 min and 10 μL of the fixed bacteria were then dropped onto a poly-l-lysine coated silicon wafer. After 30 min of drying, the bacteria were washed with water and postfixed in 4% osmium tetroxide for 1 h. After gentle washing in distilled water, cells were dehydrated through a graded series of ethanol baths (from 30% to 100%) and dried by the critical point drying (CPD) method using a Leica CPD 030. Finally, the samples were coated with a thin carbon layer using a CRESSINGTON 308R and observed with a JEOL JSM 7600F scanning electron microscope (JEOL Ltd.) at the DimaCell platform (http://www.dimacell.fr/).

Purified MVs were observed by cryogenic scanning electron microscopy (cryo-SEM) at the DimaCell platform (http://www.dimacell.fr/). Briefly, 10 μL of MVs were dropped onto a poly-l-lysine coated silicon wafer and dried for 30 min. After gentle washing in distilled water, the sample was frozen under high pressure using a high-pressure freezing system (Leica EM HPM100, Leica Microsystems Inc., Austria) before sublimation at −95°C for 5 min (Leica EM ACE900). Finally, MVs were observed with a JEOL JSM 7600F scanning electron microscope (JEOL Ltd.) operating in high-vacuum conditions at 2 kV accelerating voltage.

### Prophage induction and purification.

*L. casei* BL23 was inoculated at an OD_600nm_ of 0.1 (corresponding to 2.5 10^8^ CFU/mL) in 250 mL of fresh MRS and incubated at 37°C until the exponential phase was reached (OD_600nm_ = 0.3 to 0.4). Prophages were induced by the addition of MMC at a final concentration of 400 ng/mL. After 24 h of culture, bacteria cells and debris were removed by centrifugation at 3,000 × *g* for 10 min and filtration through a 0. 45-μm pore size filter. Phage particles were concentrated by ultrafiltration with 100K Amicon Ultra-15 centrifugal filters and purified on a Cesium chloride gradient. The phage interface was then collected and dialyzed with 50K Amicon Ultra-15 centrifugal filters against phage buffer (10 mM Tris–HCl pH 7.4, 100 mM NaCl, 10 mM MgCl_2_).

### PCR and qPCR.

To examine phage replication by PCR or qPCR, bacterial DNA were prepared using an InstaGene Matrix kit (Bio-Rad). qPCR was performed with an iTaq Universal SYBR green Supermix (Bio-Rad) in a Real-Time PCR Detection System (CFX96, Bio-Rad) following the manufacturer’s instructions. qPCRs were performed in two technical replicates and three biological replicates. Relative DNA quantity was calculated using the ΔCq method (18) after normalization by the bacterial sequence *gyrA* as an internal control. The data are presented as the fold change in DNA quantity relative to the endogenous reference *gyrA*. PCR was performed using the GoTaq DNA polymerase kit according to the recommendations of the supplier. PCR and qPCR primers are listed in Table S1.

### Reverse transcription-qPCR.

mRNA extractions were prepared using Direct-zol RNA MiniPrep (Zymo Research) followed by DNase I treatment (Roche). The absence of DNA contamination was checked by qPCR using 16 s rRNA primers. mRNAs were reverse transcribed into cDNA using High-Capacity cDNA Reverse Transcription Kits (Thermo Fisher Scientific). qPCR was performed with an iTaq Universal SYBR green Supermix (Bio-Rad) in a Real-Time PCR Detection System (CFX96, Bio-Rad). Data were analyzed by the ΔΔCt method (89) after normalization by d-lactate dehydrogenase (*ldh*) as an internal control. The data are presented as the fold change in cDNA quantity relative to the amount of *lcabl_11250* cDNA measured in the untreated parental strain (referred to as BL23). Each step was performed according to the manufacturers’ instructions. The primers are listed in Table S2.

### Confocal laser scanning microscopy.

Bacteria were washed in PBS (1×) and adjusted to an OD_600nm_ of 5 (corresponding to 1.25 10^9^ CFU/mL). Cell membranes were labeled with 4 μg/mL of FM 1–43 (Thermo Fisher Scientific) and the DNA of permeable cells were stained with 5 μM SYTOX Blue (Invitrogen) for 10 min in the dark at room temperature. Samples were spotted on microscope slides with a thin layer of 1% agarose-PBS (Sigma-Aldrich), covered by a coverslip, and samples were directly observed at room temperature at the DimaCell platform (http://www.dimacell.fr/). Images were obtained using a Leica confocal SP8 inverted microscope with a 63 × oil immersion objective lens.

### Flow cytometry.

*L. casei* BL23 strains were grown in MRS at 37°C with or without MMC at a final concentration of 400 ng/mL. MMC was added to the medium during the exponential phase (OD_600nm_ = 0.3 to 0.4). After 24 h of growth, bacteria were adjusted to 10^6^ cells/mL and permeable cells were stained with 5 μM SYTOX Blue for 10 min in the dark at room temperature. Bacteria were then analyzed by flow cytometry using a LSRFortessa Cell Analyzer (BD Biosciences) and data analyzed by R (packages flowCore and flowAI) at the Cytometrie platform (https://www.cytometrie-dijon.fr/). For each sample, a minimum of 45,000 up to 100,000 events were acquired for analysis.

### Statistical analysis.

The differences between the median values derived from the BL23 strain treated with or without MMC (Fig. 6B; Fig. S5) were analyzed with the Mann-Whitney test and *P* values lower than 0.05 were considered significant.

The Kruskal-Wallis test and the Dunn's multiple-comparison test were used to compare the BL23, DDB001, and DDB002 strains (Fig. 6A and C; Fig. 7A) and *P* values lower than 0.05 were considered significant.

A one-way ANOVA and Tukey's multiple-comparison test were used to compare qPCR (Fig. 4C; Fig. 5B; Fig. 7C; Fig. S4B; Fig. S6B) and cytometry results (Fig. S6A). *P* values lower than 0.05 were considered significant.

Error bars represent the standard deviation (SD), with * (*P* < 0.05) indicating that *P* values are lower than 0.05.

### Data availability.

Data supporting the findings of this study are available in the paper and the supplementary information files. All other data are available from the corresponding author on request.
