# Supplementary figures and images for: Spontaneous Prophage Induction Contributes to the Production of Membrane Vesicles by the Gram-Positive Bacterium Lacticaseibacillus casei BL23
Source: mBio. 2022 Oct 6;13(5):e02375-22. doi: 10.1128/mbio.02375-22 (PMC9600169; doi:10.1128/mbio.02375-22)

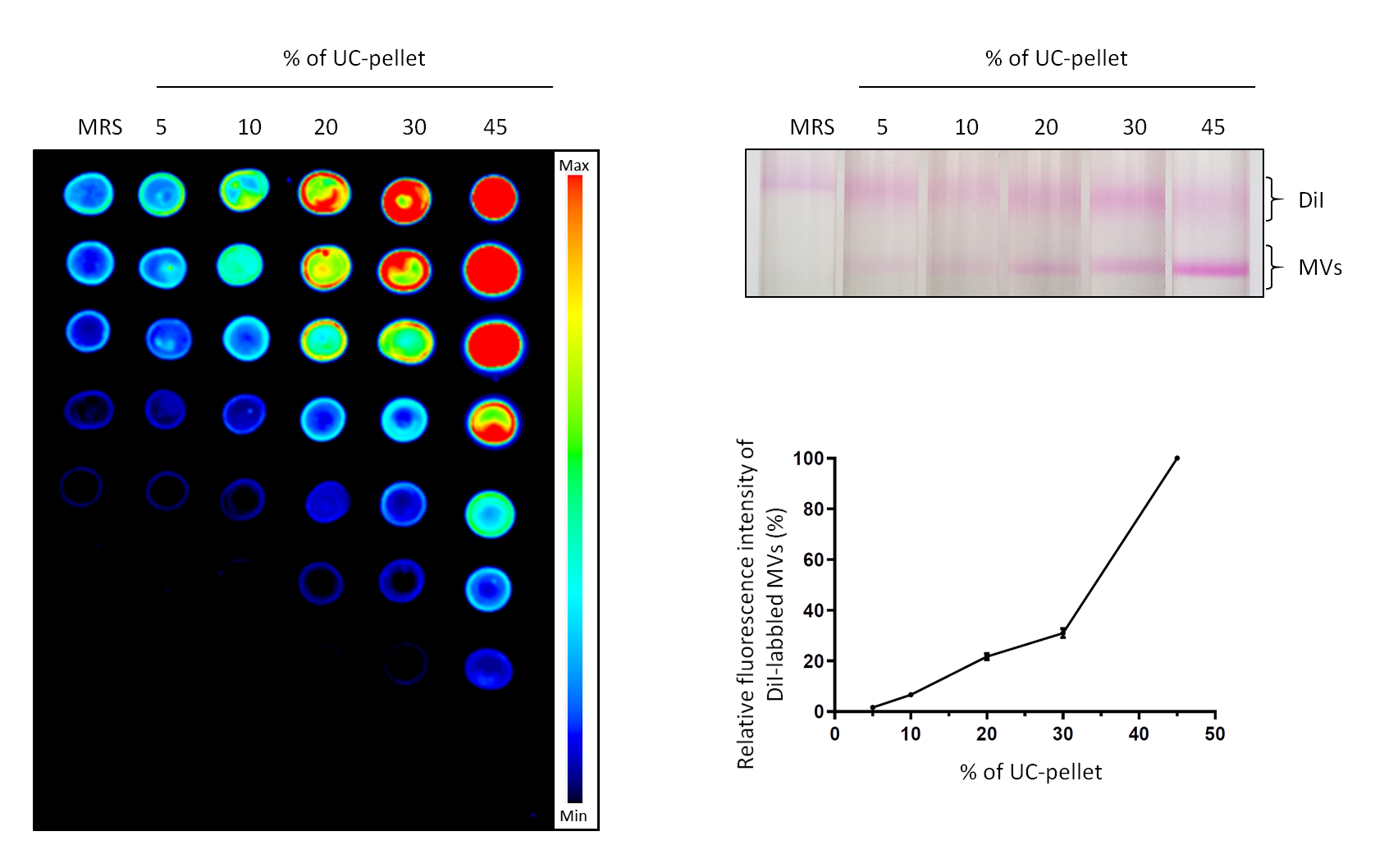

Supplement: FIG S1 [file mbio.02375-22-s0001.tif]

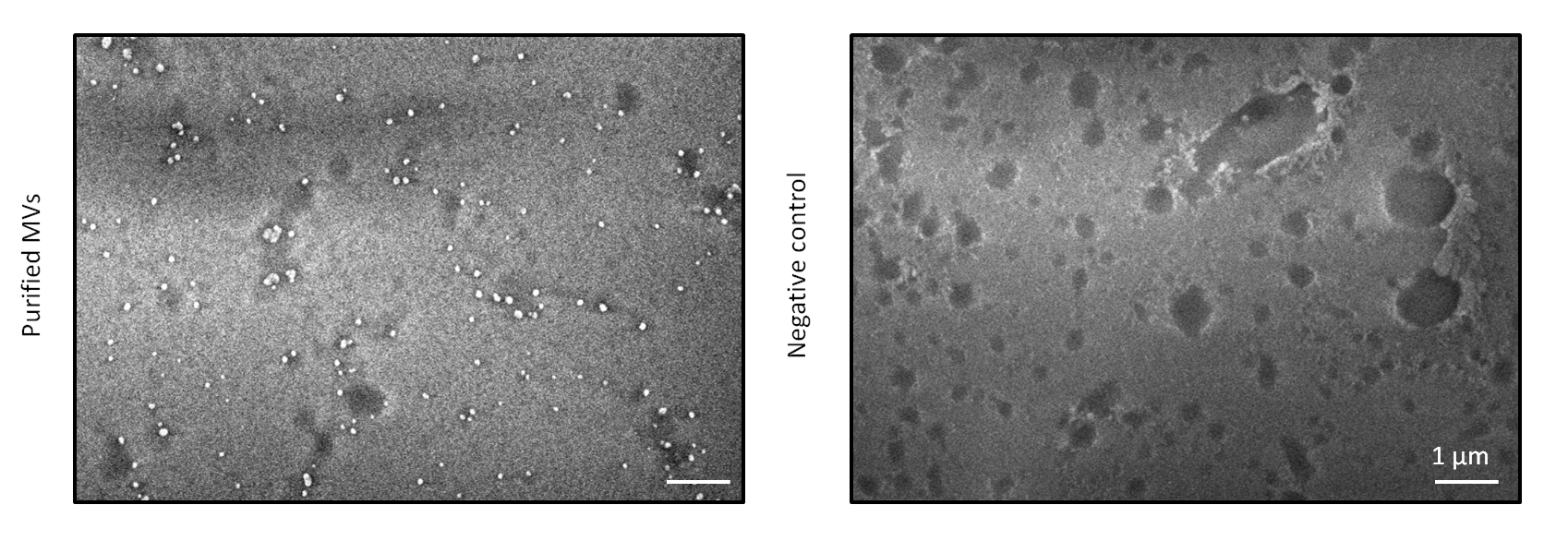

Supplement: FIG S2 [file mbio.02375-22-s0002.tif]

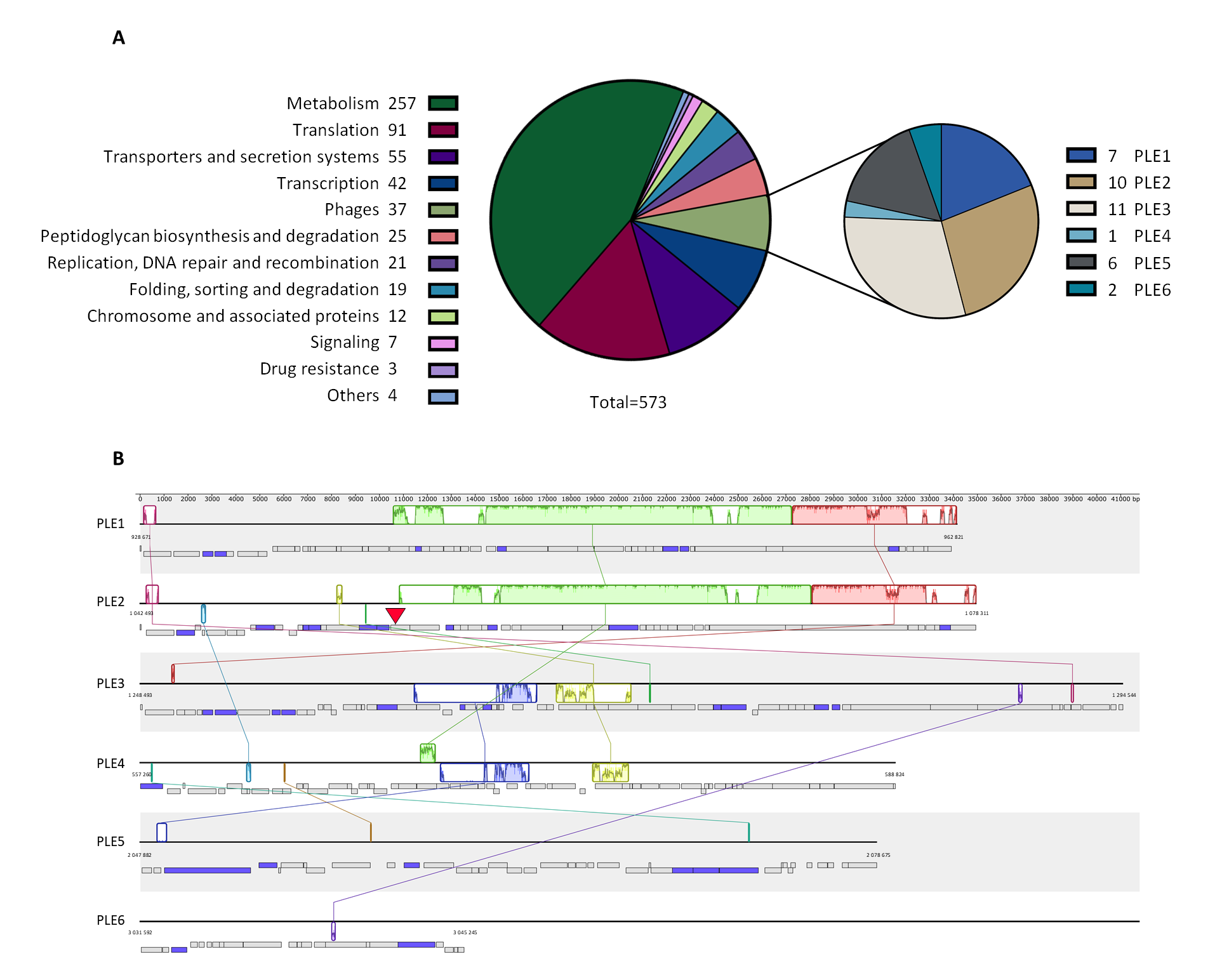

Supplement: FIG S3 [file mbio.02375-22-s0003.tif]

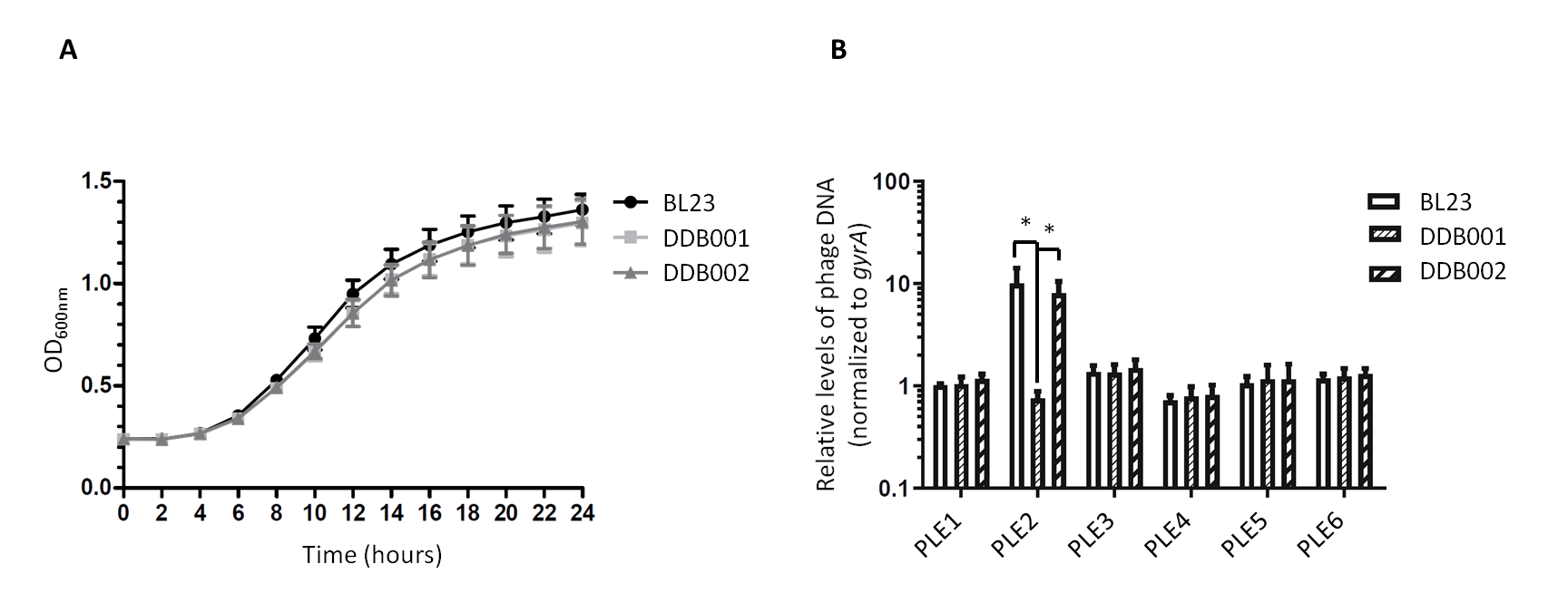

Supplement: FIG S4 [file mbio.02375-22-s0004.tif]

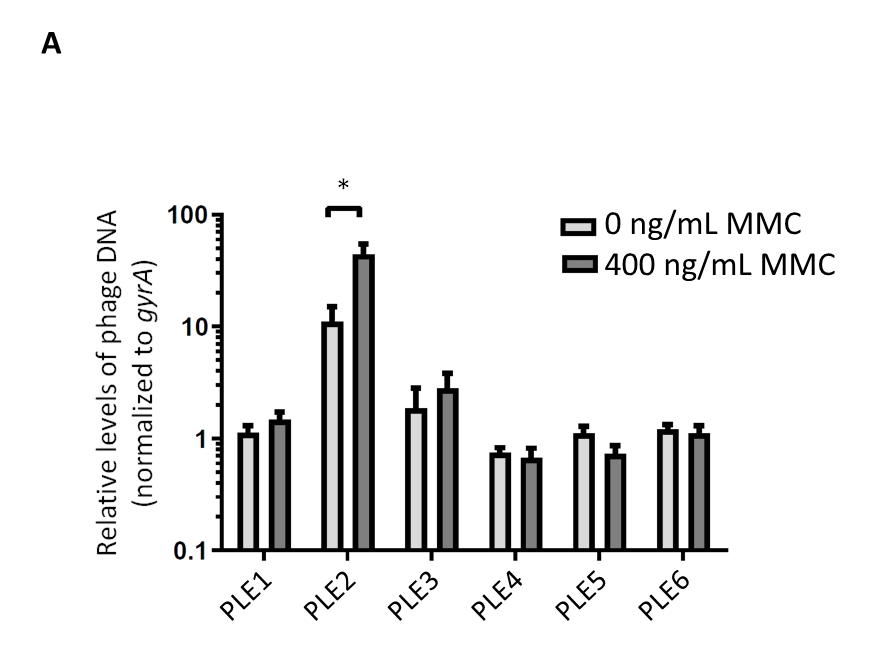

Supplement: FIG S5 [file mbio.02375-22-s0005.tif]

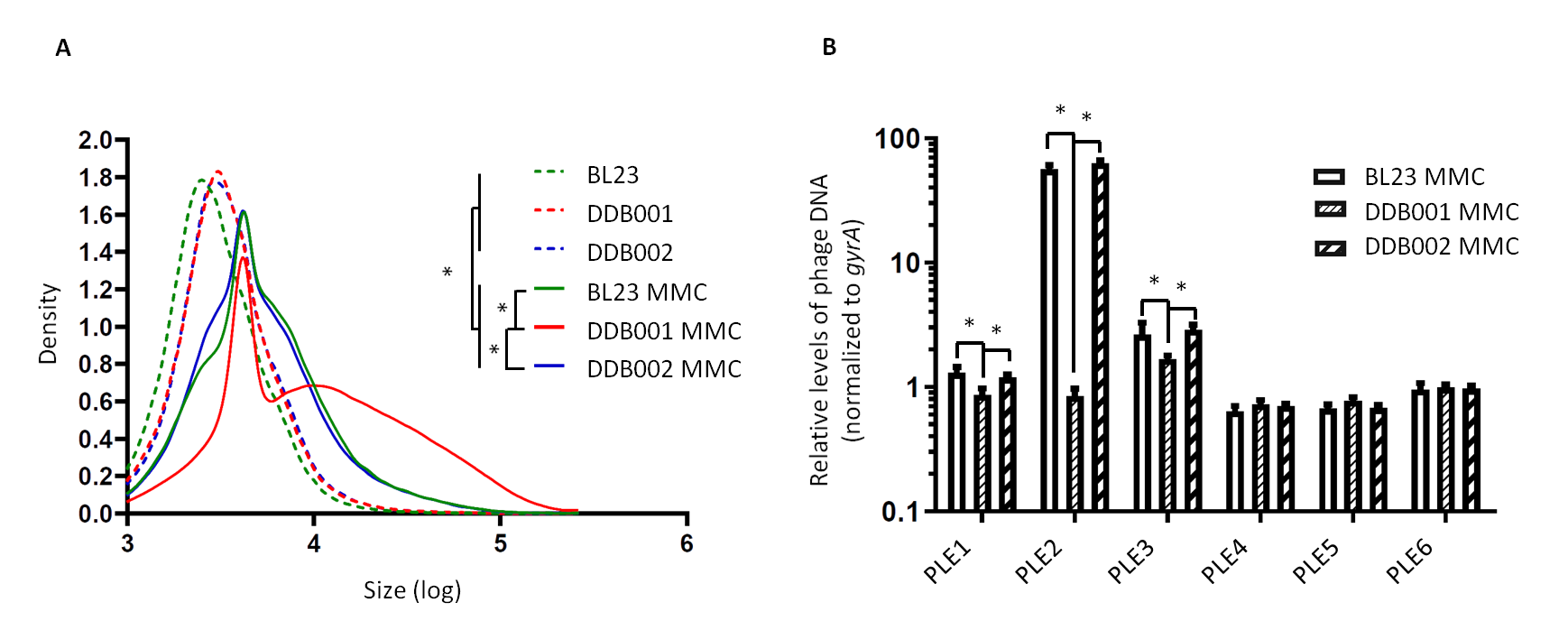

Supplement: FIG S6 [file mbio.02375-22-s0006.tif]
