# Supplementary material for: Spontaneous Prophage Induction Contributes to the Production of Membrane Vesicles by the Gram-Positive Bacterium Lacticaseibacillus casei BL23
Source: mBio. 2022 Oct 6;13(5):e02375-22. doi: 10.1128/mbio.02375-22 (PMC9600169; doi:10.1128/mbio.02375-22)
Supplement: TABLE S2 [file mbio.02375-22-s0008.docx]

**Table S2**. Strains, plasmids and oligonucleotides used in this study.

| **Strains** | **Relevant characteristic or description** | **Resistance(s)** | **Source or reference** |
| --- | --- | --- | --- |
| *Escherichia coli* |  |  |  |
| DH5α | *F^_^* Φ80*lac*ZΔM15 Δ(*lac*ZYA_*arg*F) U169 *rec*A1 *end*A1 *hsd*R17(r_k_^_^, m_k_^+^) *pho*A *sup*E44 *thi*_1 *gyr*A96 *rel*A1 λ^_^ |  | (1) |
| *Lacticaseibacillus casei* |  |  |  |
| BL23 | Laboratory strains; sequenced genome |  | CECT 5275 |
| DDB001 | BL23 *gp271*(*lcabl_10980*)::pRV300 | Ery^r^ | This work |
| DDB002 | BL23 containing pRV002 inserted into the genome at the position 2,441,492 | Ery^r^ | This work |

| **Plasmids** | **Relevant characteristic or description** | **Resistance(s)** | **Source or reference** |
| --- | --- | --- | --- |
| pRV300 | Insertional vector for lactobacilli | Amp^r^, Ery^r^ | (2) |
| pRV001 | pRV300 with a 435 bp fragment of the gene *lcabl_10980* cloned at EcoRI site | Ery^r^ | This work |
| pRV002 | pRV300 with a 442 bp fragment of an intergenic region (comprised between the genes *lcabl_24810* and *lcabl_24820*) cloned at HindIII/SacI sites | Ery^r^ | This work |

Ery^r^, erythromycin resistant; Amp^r^, ampicillin resistant; CECT, Colección Española de Cultivos Tipo.

| **Oligonucleotides** | **Sequence (5’_3’)** |
| --- | --- |
| Construction of pRV001 and pRV002 | |
| *P001_FD* | ACTAATGAATTCGGCTTCTATGTGTTACCAATGC |
| *P001_RV* | CCTAAGGAATTCTAATCCATCACTGGCTTCAAC |
| *P002_FD* | GCACACAAGCTTTGCCAAACTCCGCAATCTCC |
| *P002_RV* | AGTCTTGAGCTCTTTTACGCTCTATGACACGCAC |
| qPCR primers | |
| Quantification of the 6 predicted prophage sequences | |
| *PLE1_FD* | AGTGCTGGATCGGTGGTCAAAG |
| *PLE1_RV* | ACGCCTTGAAGTTGCTGACTG |
| *PLE2_FD* | CAACCGATCAACCTGCTACACC |
| *PLE2_RV* | GATTATCCATGCCTGCCGCTCC |
| *PLE3_FD* | CGTTGGTCACCGCTCTCATTG |
| *PLE3_RV* | CCCAGACAGCTTTAGCAAGACC |
| *PLE4_FD* | CTTCACCACGGGCAAACATCTG |
| *PLE4_RV* | TTGCTTGCTCTGTGTGGCTTAC |
| *PLE5_FD* | ACTGACGGCATCACAAGTGTTC |
| *PLE5_RV* | TGGCAAACCAAGCAGCGTAC |
| *PLE6_FD* | CTTGATGGCGTGCTGGTATTCG |
| *PLE6_RV* | TCCACCGTTTGGCGTTGTTTC |
| Quantification of circularized PLE2 DNA | |
| *PLE2_attP_FD* | ATTCTTTTTCCCTACTTTTCCG |
| *PLE2_attP_RV* | AAGTCCTCTGCTCGCTAACG |
| RT-qPCR primers | |
| Quantification of holin-endolysin systems of PLE1, PLE2, PLE3 | |
| *PLE1_HOL_FD* | AACGGTAGCGGCAGTCTTC |
| *PLE1_HOL_RV* | GTAAGCTAACGCCTGCTGG |
| *PLE1_ENDO_FD* | TGTATGGCTCTCGTAACGGTGC |
| *PLE1_ENDO_RV* | TGCCTAAGCTCACAGTGGATGG |
| *PLE2_HOL_1_FD* | ACAGTTAGCGGTGACCAATCG |
| *PLE2_HOL_1_RV* | AGCACGAAGGCTGTTATGATCG |
| *PLE2_HOL_2_FD* | CGTAACGGCGGCAGAACATC |
| *PLE2_HOL_2_RV* | TCTGCTCGTCTGTGCTGGTG |
| *PLE2_ENDO_FD* | GCTGCTCGATATGGCATTCC |
| *PLE2_ENDO_RV* | ACGGATCAGTGTGGTCACCC |
| *PLE3_HOL_FD* | CGGCAGCTTTGTTGGTTGTAC |
| *PLE3_HOL_RV* | CTCTGTGGTTGGGTCAACGG |
| *PLE3_ENDO_FD* | GGATTGGGCGAAGTATCAGGG |
| *PLE3_ENDO_RV* | GCCTTGGCACTAGCAATTTGG |
| qPCR internal control | |
| *LDH_FD* | CACCGTTGAATGGGCTAAAGG |
| *LDH_RV* | GAATCGTCAGGAACTTGATACC |
| *RNA16S_FD* | AGAGTTTGATCCTGGCTCAG |
| *RNA16S_RV* | CCACTCGTTCCATGTTGAATCTC |
| *GYRA_FD* | AACCGTACAAGAAGAGTGC |
| *GYRA_RV* | AGTCCTGTGCCATTCGTAC |
| PCR primers | |
| Amplification of the attachment-site sequences | |
| *PLE2_1* | AGACACGTCGCAAGATGAAC |
| *PLE2_2* | AATGAACGGTGAAGATCGTCG |
| *PLE2_3* | TGAGATGAAGCAACGAGACGAC |
| *PLE2_4* | CGAGTTTGCCACAGTACACAGAC |

FD, forward; RV, reverse

**REFERENCES**

1. Taylor RG, Walker DC, McInnes RR. 1993. *E. coli* host strains significantly affect the quality of small scale plasmid DNA preparations used for sequencing. Nucleic Acids Res 21:1677–1678.

http://dx.doi.org/10.1093/nar/21.7.1677.

2. Leloup L, Ehrlich SD, Zagorec M, Morel-Deville F. 1997. Single-crossover integration in the *Lactobacillus sake* chromosome and insertional inactivation of the ptsI and lacL genes. Appl Environ Microbiol 63:2117–2123. http://dx.doi.org/10.1128/aem.63.6.2117-2123.1997.
